# Genetic Encoding of Pentafluorophosphato‐Phenylalanine Provides PF_5_‐Proteins as Phosphoprotein Mimetics

**DOI:** 10.1002/anie.202518789

**Published:** 2025-11-14

**Authors:** Anna Magdalena Ambros, Haocheng Qianzhu, Markus Tiemann, Edan Habel, Katrin Denzinger, Hana Zupan, Matteo Accorsi, Bettina G. Keller, Gerhard Wolber, Thomas Huber, Jörg Rademann

**Affiliations:** ^1^ Department of Biology Chemistry, Pharmacy Institute of Pharmacy Freie Universität Berlin Königin‐Luise‐Str. 2+4 14195 Berlin Germany; ^2^ Research School of Chemistry Australian National University Australian Capital Territory 2601 Australia; ^3^ Department of Biology Chemistry, Pharmacy Institute of Chemistry Freie Universität Berlin Arnimallee 22 14195 Berlin Germany

**Keywords:** Genetic encoding, Non‐canonical amino acids, Pentafluorophosphates, Phosphotyrosine mimetics, Protein tyrosine phosphatases

## Abstract

Protein phosphorylation is one of the most important posttranslational modifications altering the structure, stability, and activity of more than 13 000 human proteins. In this work, the phosphotyrosine mimetic pentafluorophosphato‐difluoromethyl‐phenylalanine (PF_5_CF_2_Phe) was genetically encoded and incorporated into three different proteins. Screening two libraries of orthogonal aminoacyl‐tRNA synthetases identified enzymes enabling the efficient and specific incorporation of PF_5_CF_2_Phe into red fluorescent protein (RFP) via amber stop codon suppression. Two model proteins, human ubiquitin (Ubq) and the B1 immunoglobulin‐binding domain of streptococcal protein G (GB1), were prepared with PF_5_CF_2_Phe mutations and investigated for potential interaction partners. While native GB1 showed no binding to protein tyrosine phosphatases (PTP), PF_5_‐GB1, with PF_5_CF_2_Phe at position 17, was a strong inhibitor of the phosphatases PTP1B and SHP2. PF_5_‐Ubq was produced and converted into the first example of a protein carrying the most prominent phosphotyrosine mimetic, phosphono‐difluoromethyl phenylalanine (PO_3_CF_2_Phe). With increasing need in the biosciences to delineate the functions of complex phosphorylation patterns, genetic encoding of PF_5_CF_2_Phe yielding phosphoprotein mimetics opens unique opportunities for precise functional studies where site‐specific and homogeneous protein modifications are required.

## Graphical abstract

Genetic code expansion with a unique hyper‐fluorinated phosphotyrosine analog. In this work, we successfully incorporated the unnatural amino acid pentafluorophosphato‐difluoromethyl‐phenylalanine, carrying seven fluorine atoms and a permanent negative charge into three different proteins via the use of mutated orthogonal aminoacyl‐tRNA synthetases. Biological testing revealed the great potential of this approach for furnishing functional phosphoprotein mimetics.

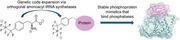

Post‐translational modifications of proteins are fundamentally important for the regulation of protein structure and function in higher organisms. Protein tyrosine phosphorylation is an especially interesting modification considering the prominent roles of phosphotyrosine residues in membrane receptors, proteins of signaling cascades, and transcription factors.^[^ ^1^, ^2^, ^3^ ^]^ Protein phosphorylation is a delicately dynamic process, and phosphoproteins are constantly modified in response to cues in living cells. This leads to heterogeneity of phosphorylation, which is difficult to control in vivo, and when phosphoproteins are isolated, is even further exacerbated due to the labile nature of the phosphoester bond. In consequence, chemically stable phosphoprotein mimetics are required to study effects of protein phosphorylation. One approach to obtain proteins with stable phosphotyrosine analogs is to introduce a non‐canonical amino acid (ncAA) acting as a biomimetic of phosphotyrosine biomimetic by genetic code expansion (GCE). This method has made it possible to precisely install functional groups in proteins to probe their structural and functional consequences in vitro and in cells.^[^ ^4^, ^5^, ^6^, ^7^, ^8^, ^9^ ^]^ Genetic encoding of phosphotyrosine **1** (pTyr) has proven to be difficult because of its instability and low cellular uptake.^[^ ^10^, ^11^, ^12^ ^]^ Non‐cleavable phosphotyrosine mimetics like phosphonates overcome the instability issue, but they are still highly charged, limiting membrane permeability.^[^ ^12^ ^]^ While 4‐phosphonomethyl‐phenylalanine (PO_3_CH_2_Phe)^[^ ^12^ ^]^ and carboxymethyl phenylalanine^[^ ^13^, ^14^ ^]^ were incorporated into proteins, no proteins with the most relevant phosphotyrosine mimetic, 4‐phosphono‐difluoromethyl‐phenylalanine **2** (PO_3_CF_2_Phe)^[^ ^15^, ^16^, ^17^ ^]^ have been reported yet.

Recently, we have discovered the pentafluoro‐phosphates 4‐pentafluorophosphato‐difluoromethyl‐phenylalanine **3** (PF_5_CF_2_Phe) and 4‐pentafluorophosphato‐carboxy‐phenylalanine **4** (PF_5_COPhe) as novel, physiologically stable phosphotyrosine mimetics.^[^ ^18^, ^19^ ^]^ Both PF_5_‐phenylalanines were stronger inhibitors of the protein tyrosine phosphatase PTP1B than phosphonate **2** and could be readily incorporated into peptides using solid‐phase peptide synthesis (SPPS). Pentafluorophosphates display a unique expansion of the chemical space, combining a steady negative charge and polarity with remarkable hydrophobicity, leading to fluorine‐specific interactions with the protein pocket and the water phase and suggesting a potential for membrane permeability.^[^ ^20^ ^]^ These considerations prompted us to investigate the genetic encoding of pentafluorophosphate **3** and its incorporation into proteins.

To study the differences in charge and hydrophobicity of the pTyr analogs on an atomic level, we employed density functional theory calculations. The energies of N‐acetyl‐amino acid amides of **1**–**3** were minimized in implicit water and benzene, respectively. Mulliken atomic charges in water show the broad distribution of negative charge of the PF_5_ group (−0.35 per fluorine atom) over a larger Van der Waals surface area (113.40 Å^2^ for CF_2_PF_5_^−^) whereas the phosphate and phosphonate exhibit a more concentrated negative charge on the oxygen atoms (−0.8 per oxygen atom) and smaller surface areas (81.71 Å^2^ for O‐PO_3_^2−^ and 98.03 Å^2^ for CF_2_PO_3_^2−^) (Tables S1–S3). The PF_5_‐structure **Ac‐3‐NH_2_** furthermore exhibits substantially lower energy in an apolar benzene environment, which highlights its increased hydrophobicity compared to phosphate and phosphonate (Figures 1 and S1).

**Figure 1:**
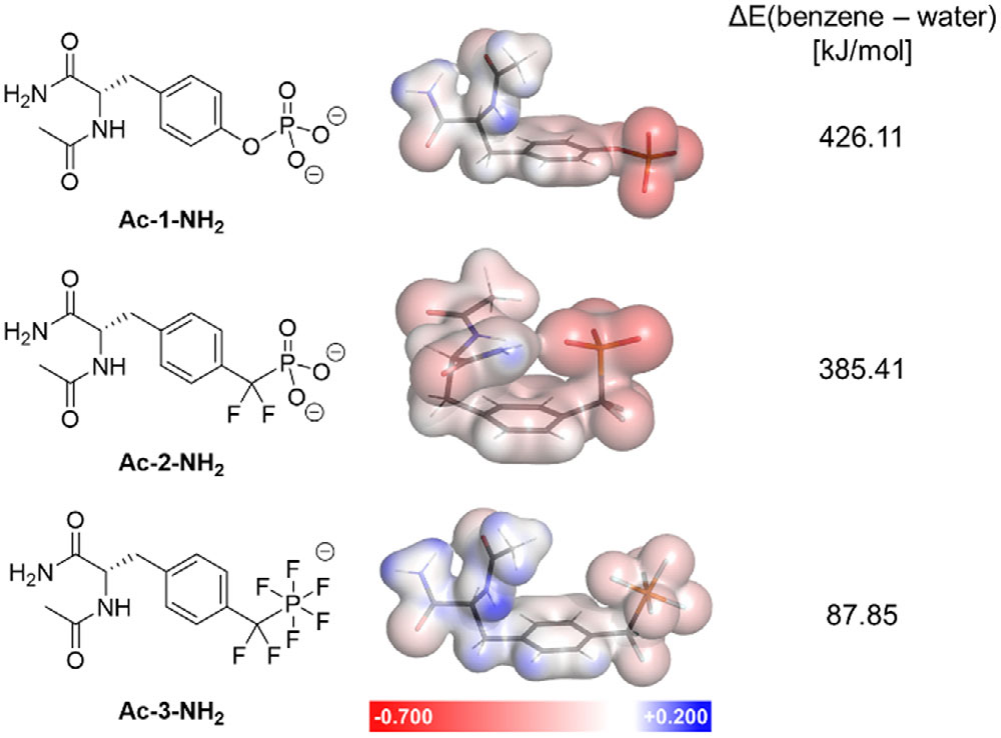
Structures, DFT‐derived electrostatic potential surfaces in implicit water, and energy differences between implicit benzene and water of N‐acetyl‐amides of phosphotyrosine (pTyr) **1** and its biomimetics PO_3_CF_2_Phe **2** and PF_5_CF_2_Phe **3**.

Prior to genetic encoding, the synthesis of unprotected amino acid **3** was reconsidered (Figure 2a). Previously, pentafluorophosphates were prepared from phosphonate **5** in a protocol via phosphoryl halogenides treated with anhydrous tetramethyl ammonium fluoride, producing high amounts of salts as by‐products during aqueous work‐up.^[^ ^18^, ^19^ ^]^ Systematic investigation of alternative fluorination conditions revealed that neat saturated HF:pyridine complex (Olah's reagent) converted phosphonate **5** smoothly into pentafluorophosphato‐carbonyl product **7** (Fmoc‐**4**‐OMe) in 75% yield, involving hydrolysis of the benzylic difluoromethyl carbon to a carbonyl moiety. Quenching of HF with trimethylsilyl methyl ether (TMSOMe) yielded volatile, hardly toxic trimethylsilyl fluoride and facilitated the work‐up considerably, avoiding the use of water. Treating **5** with a combination of Olah´s reagent and diethylamino sulfur trifluoride (DAST) provided **6** in a single step. Saponification of the methyl ester in **6** and Fmoc‐cleavage yielded pentafluorinated amino acid **3**, used for amber stop codon suppression.

**Figure 2:**
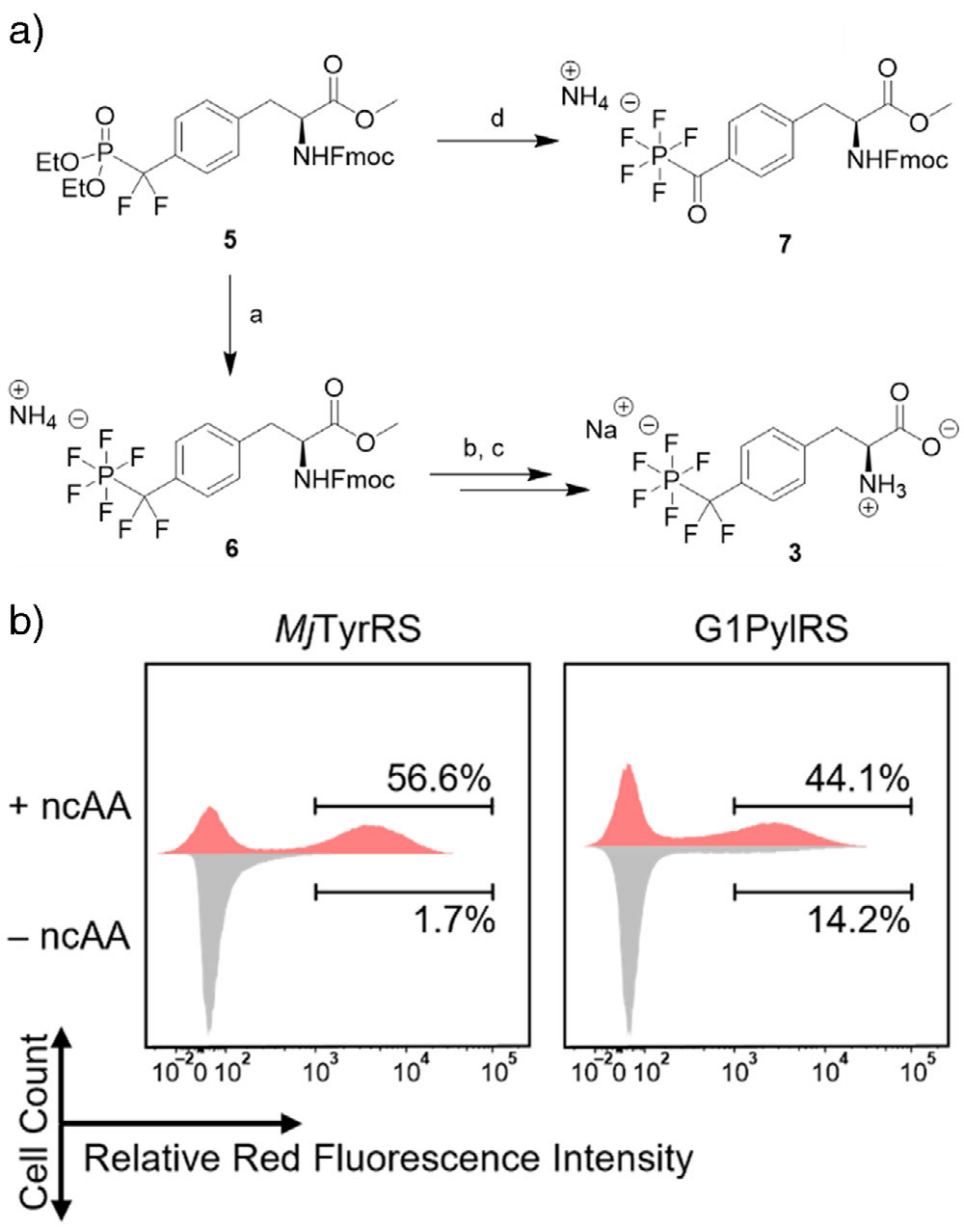
a) Improved synthesis of PF_5_CF_2_Phe **3** and Fmoc‐PF_5_COPhe‐OMe **7**; Reaction conditions: a) 50 eq. HF:pyridine (70:30 wt%), 5 eq. DAST, 50 °C, after 7 h quenching with TMSOMe until pH = 7–8, 55%; b) protease from *B. licheniformis* in 10 mM NH_4_HCO_3_ buffer stirring overnight, 96%; c) 20% piperidine in acetonitrile 2 h, Amberlite^TM^ IRC‐120 Na^+^ form, 97%; d) 50 eq. HF:pyridine (70:30 wt%), 50 °C, after 4 h quenching with TMSOMe until pH = 7–8, 75%; b) Histograms from the final rounds of fluorescence‐activated cell sorting (FACS) selection to identify *Mj*TyrRS and G1PylRS enzymes active toward **3**. The horizontal axis reports the red fluorescence intensity from expression of the mRFP1 gene, which is interrupted by an amber stop codon. The vertical axis represents the cell count, with the red fluorescence of cells grown with ncAA plotted in the positive direction (red) and the fluorescence measured without ncAA plotted in the negative direction (gray). The difference indicates the presence of ncAA‐specific RS enzymes in the remaining gene pool.

To identify functional PF_5_CF_2_Phe‐specific aminoacyl‐tRNA synthetases (PFRS) for protein expression, two distinct orthogonal translation systems (OTS) were employed, the *Methanocaldococcus jannaschii* tyrosyl‐tRNA synthetase/tRNA^Tyr^ pair (*Mj*TyrRS), previously used in bacterial systems for para‐pentafluorosulfanyl phenylalanine (SF_5_Phe) incorporation,^[^ ^21^ ^]^ and the methanogenic archaeon ISO4‐G1 pyrrolysyl‐tRNA synthetase/tRNA^Pyl^ pair (G1PylRS), compatible with eukaryotic expression and reportedly suitable for incorporating pTyr analogs.^[^ ^11^ ^]^ This allowed us to directly compare the two OTS systems for the incorporation of the negatively charged PF_5_‐amino acid **3**.

The selection of PFRS enzymes was conducted in *E. coli* DH10B cells co‐transformed with the selection plasmid pBAD‐H6RFP, which carries the gene of mRFP1 red fluorescent protein preceded by an N‐terminal His‐tag and the amber stop codon TAG, and the tRNA synthetase plasmid pBK‐*Mj*RS containing the *Mj*TyrRS library or pBK‐G1RS harboring the G1PylRS library.^[^ ^21^, ^22^ ^]^

Six sites in the substrate binding pocket of the *Mj*TyrRS and seven sites in G1PylRS were selectively mutated or fully randomized to generate the tRNA synthetase libraries (Table S4). Cells were subjected to alternating rounds of positive and negative selection using fluorescence‐activated cell sorting (FACS). In positive selection rounds, cells were cultured in lysogeny broth (LB) medium supplemented with 1 mM PF_5_CF_2_Phe **3**. Cells exhibiting high levels of red fluorescence, indicative of amber codon readthrough, were collected. In negative selection rounds, cells were cultured without the ncAA, and those with low red fluorescence were selected to eliminate false‐positive tRNA synthetase variants that recognize natural amino acids. This alternating selection strategy allowed for the enrichment of mutants that specifically incorporate **3** (Figures 2b, S5, and S6).

In the *Mj*TyrRS selection, enrichment of a functional RS population appeared after the third round (Figures 2b and S5), where significantly more high‐red‐fluorescent cells were observed in the presence (56.6%) than in the absence of the ncAA (1.7%). When screening the G1PylRs library, a similar population emerged after the fifth round (44.1%:14.2%) (Figures 2b and S6). To demonstrate the reproducibility of the expression level of the selection marker in our method, 120 monoclonal cells from both 5% (top 5%) and 56.6% (total positive) of the *Mj*‐3P + sample with the highest red fluorescence level were individually characterized (Figure S7). As expected, the fluorescence level from cells in the top 5% set was substantially higher than in the total positive set, at 1.7 times higher on average, indicating that single‐cell fluorescent intensities from FACS correlated well with function and the best candidates are found in the top 5% fraction of the final positive selection. Seven best candidates from the *Mj*‐top‐5% set were sequenced (Table S4), showing high sequence diversity. The top 5% fraction of G1‐5P + sample was also individually characterized (Figure S8). 15 final candidates were sequenced and showed a high level of convergence (Tables S4 and S5).

Notably, the most active G1PylRS mutants consistently contained a tryptophan to arginine mutation at position 237, which is, according to a computational model, in close proximity to the negatively charged PF_5_ moiety (Figure S9) and seems to be important to selectively bind PF_5_CF_2_Phe at the amino acid recognition site of the G1 tRNA synthetase. Comparison of both OTS mutants expressed on the same plasmid backbone showed overall higher efficiency and specificity of G1PylRS variants (Figures S7 and S8).

Despite the native affinity of *Mj*TyrRS for tyrosine, the engineered G1PylRS variants outperformed *Mj*TyrRS mutants in incorporating PF_5_CF_2_Phe, highlighting the tunability and broad substrate scope of the PylRS scaffold for aromatic ncAAs. Next, substrate specificity of the seven selected synthetase candidates for PF_5_CF_2_Phe was explored in comparison to other tyrosine derivatives with negatively charged bulky substituents in the *para* position. Sulfotyrosine (sTyr), 4‐phosphonomethyl‐phenylalanine (PO_3_CH_2_Phe), and 4‐phosphono‐difluoromethyl‐phenylalanine (PO_3_CF_2_Phe) were tested as potential substrates (Figure S10). Amber codon readthrough did not occur when 1 mM of PO_3_CH_2_Phe or PO_3_CF_2_Phe was supplied to the medium. Interestingly, sTyr was not incorporated via any of the *Mj*TyrRSs, whereas several G1PylRSs recognized sTyr with up to 15% of the incorporation efficiency observed for PF_5_CF_2_Phe. Based on our previous experience, increasing glycine concentration in the culture medium to 20 mM modestly enhanced sTyr incorporation, potentially due to increased uptake (Figure S11A).^[^ ^23^, ^24^ ^]^ We explored whether this strategy could also be implemented for PF_5_CF_2_Phe. Glycine supplementation, however, had no positive effect on PF_5_CF_2_Phe uptake but led to a reduction in protein yield (Figure S11B). This contrasting response suggests that PF_5_CF_2_Phe and sTyr rely on distinct import mechanisms, and the amphiphilicity of PF_5_CF_2_Phe may favor its diffusion through the unaltered outer membrane independent of glycine treatment.

From the culture samples for testing PF_5_CF_2_Phe incorporation, His_6_‐PF_5_CF_2_Phe‐mRFP red fluorescent protein (PF_5_‐RFP) was isolated by using a Ni‐NTA column and identified by mass spectrometry, showing the deconvoluted mass at 26 547.9 Da (expected mass 26 545.7 Da) together with signals for proteins losing one or two molecules of HF (Figure S12). To increase the protein yield achieved through GCE, the best‐performing G1PyIRS variant, G1PF16, was cloned into the pRSF vector.^[^ ^23^, ^24^ ^]^ Two proteins, human ubiquitin (Ubq) and the B1 immunoglobulin‐binding domain of streptococcal protein G (GB1), were selected for subsequent experiments. We previously used these model proteins to validate genetic code expansion, and they allowed us to investigate incorporation efficiencies relative to other ncAAs. Ubq and GB1 have the additional advantages of small size and high solubility, and their structures are well characterized. PF_5_CF_2_Phe was incorporated into permissive, solvent‐exposed sites (Glu18 for Ubq and Thr17 for GB1, Figure 3a). *E. coli* B95.ΔAΔ*fab* cells cotransformed with pRSF‐G1PFRS and the pCDF plasmid encoding the protein of interest were used for expressing PF_5_CF_2_Phe‐incorporated proteins.^[^ ^25^ ^]^ Protein yields were quantified, and incorporation fidelity of PF_5_CF_2_Phe was confirmed by mass spectrometry (Figure S12).

**Figure 3:**
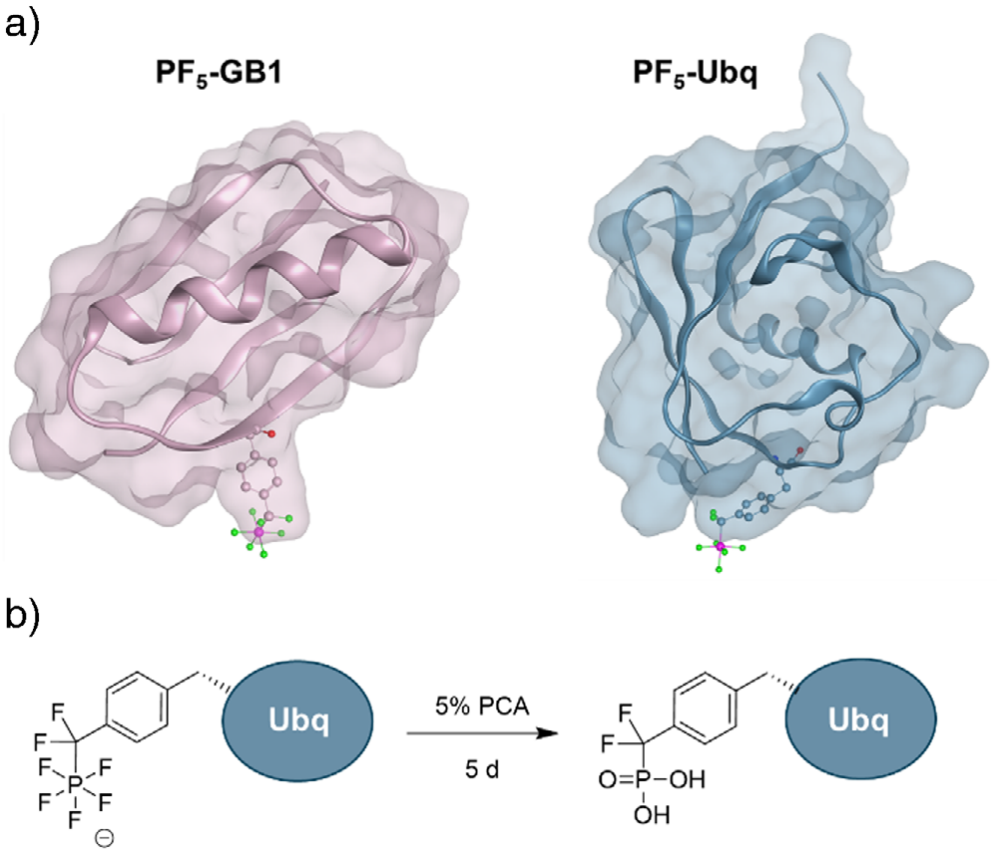
a) Protein models of PF_5_‐GB1 and PF_5_‐Ubq were built from RCSB entries 2J52 (GB1) and 1UBQ (Ubq). PF_5_CF_2_Phe was incorporated at the respective mutation sites with MOE residue and minimized with the Amber14 force field. b) PF_5_‐Ubq was hydrolyzed to the corresponding phosphonate PO_3_‐Ubq by incubating it with 5% perchloric acid (PCA) for 5 d.

Having the first PF_5_‐proteins in our hands, we wondered if it was possible to convert the PF_5_CF_2_Phe side chain to the important phosphotyrosine mimetic, phosphono‐difluoromethyl phenylalanine **2**.^[^ ^15^, ^16^, ^17^ ^]^ Incorporation of PO_3_CF_2_Phe via genetic encoding and evolution of a suitable tRNA‐synthetase has been unsuccessful so far, possibly due to the polarity and the two negative charges of this amino acid at pH 7.

When a solution of PF_5_‐Ubq was treated with 5% perchloric acid at 4 °C for 5 days, the protein was converted quantitatively to its H_2_PO_3_CF_2_‐derivative PO_3_‐Ubq (Figure 3b). Precipitation in trichloroacetic acid and dissolution in buffer yielded the protein PO_3_‐Ubq in pure form, which was identified in protein MS (Figure S13). Thus, hydrolysis of PF_5_‐proteins containing the phosphostyrosine mimetic **3** constitutes the first access to PO_3_‐CF_2_‐proteins.

Both PF_5_‐proteins and the PO_3_‐protein were investigated in enzyme activity assays of the protein tyrosine phosphatases PTP1B and SHP2 using 6,8‐difluoro‐4‐methylumbelliferyl phosphate (DiFMUP) as a fluorogenic substrate.^[^ ^18^, ^19^ ^]^ Initial testing showed unexpected intrinsic phosphatase activity of the Ni‐NTA PF_5_‐protein samples, indicating the presence of a foreign phosphatase as impurity in the samples. This was confirmed by incubating the samples with known phosphatase inhibitors, with sodium pervanadate (Na_3_VO_4_) completely abolishing the activity (Figure S14). Pull‐down experiments followed by peptide mass analysis after tryptic digest (Tables S6 and S7) identified the enzyme histidine biosynthesis bifunctional protein (HisB) in the protein samples. HisB exerts phosphatase activity, cleaving L‐histidinol phosphate as its native substrate,^[^ ^26^ ^]^ and is a member of the human HAD phosphatase family, for which DiFMUP has been reported as a generic substrate.^[^ ^27^ ^]^ Possibly, an affinity of HisB for the PF_5_‐proteins has contributed to the enrichment of phosphatase activity in the protein samples. Additional purification by high‐resolution size exclusion chromatography (SEC) led to complete removal of the phosphatase activity in the protein samples (Figures S15 and S16). Subsequently, the activity of the PF_5_‐proteins and their native analogs was tested against PTP1B and SHP2 (Figure 4). PF_5_‐GB1 was a potent inhibitor of both PTP1B and SHP2 with about 10% residual activity, much stronger than the amino acid alone, while the native GB1 protein did not show any effect on the enzymes (Figure 4).

**Figure 4:**
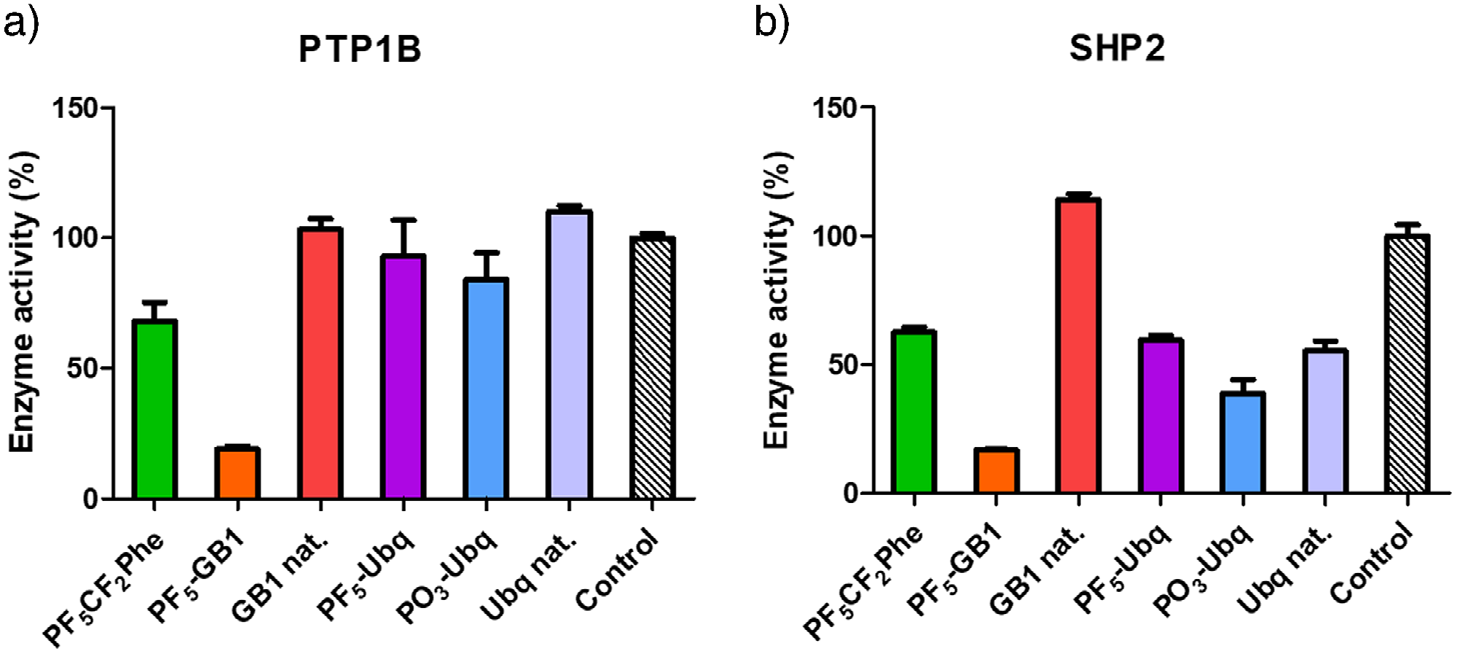
Phosphatase activity assay results; a) PTP1B showed a residual activity of 68% after incubation with the amino acid PF_5_CF_2_Phe alone; PF_5_‐GB1 showed a strong phosphatase inhibition with 19% rest activity compared to 103% for native GB1; the phosphonate‐carrying Ubq protein had only little effect on PTP1B with 84% residual activity for PO_3_‐Ubq and 93% for PF_5_‐Ubq; native Ubq did not show any inhibitory effect with 110% activity; b) SHP2 exhibited similar effects for the amino acid PF_5_CF_2_Phe with a rest activity of 63%. PF_5_‐GB1 was also a strong inhibitor of SHP2 with a residual activity of 17% compared to native GB1 with 114%. Surprisingly all three Ubq proteins resulted in around 50% decrease in activity with 59% residual activity for PF_5_‐Ubq, 39% for PO_3_‐Ubq, and 55% for native Ubq. All compounds were tested at 25 µM after an incubation time of 15 min.

In contrast, PF_5_‐Ubq, PO_3_‐Ubq, and native Ubq did not significantly inhibit PTP1B, while the amino acid PF_5_CF_2_Phe alone reduced the activity to 63%. In the case of SHP2, however, both native ubiquitin and PF_5_‐Ubq showed around 50% inhibition, and PO_3_‐Ubq was a slightly more active inhibitor. While the inhibition of SHP2 by ubiquitin has not been reported yet, it is known that full‐length SHP2 forms an auto‐inhibited, closed conformation in which the N‐SH2 domain of SHP2 binds to the PTP domain, blocking the active site of the enzyme.^[^ ^28^, ^29^ ^]^ Since our assay was conducted with a truncated version of the protein only containing the catalytically active PTP domain, it could be possible that Ubq binds in place of the N‐SH2 domain, effectively inhibiting the phosphatase activity.

To rationalize the obtained biological data, protein–protein docking experiments were conducted. Since pentafluorinated residues are not represented by the Amber14 force field, GB1 and Ubq were first docked to the catalytic domains of PTP1B and SHP2 in their native form. Docking results were manually filtered for poses showing interactions in proximity to the active site of the enzymes. In these poses, PF_5_CF_2_Phe was incorporated at the respective mutation sites (Thr17 for GB1, Glu18 for Ubq) and minimized using the standard Amber14 force field.

Native GB1 fitted well to the protein surface of PTP1B; however, it did not show any interactions with the catalytic site of the phosphatase (Figure S18A). In PF_5_‐GB1, however, the PF_5_‐residue reached into the narrow binding pocket of PTP1B, anchoring the protein through interactions with Arg221 and Cys215 (Figure 5a). Likewise, native GB1 did not show interactions with the catalytic site of SHP2 (Figure S18C), while PF_5_‐GB1 inserted the PF_5_CF_2_Phe residue into the binding pocket of SHP2, forming a salt bridge with Arg465, giving a plausible explanation for the observed biological activities (Figure 5b).

**Figure 5:**
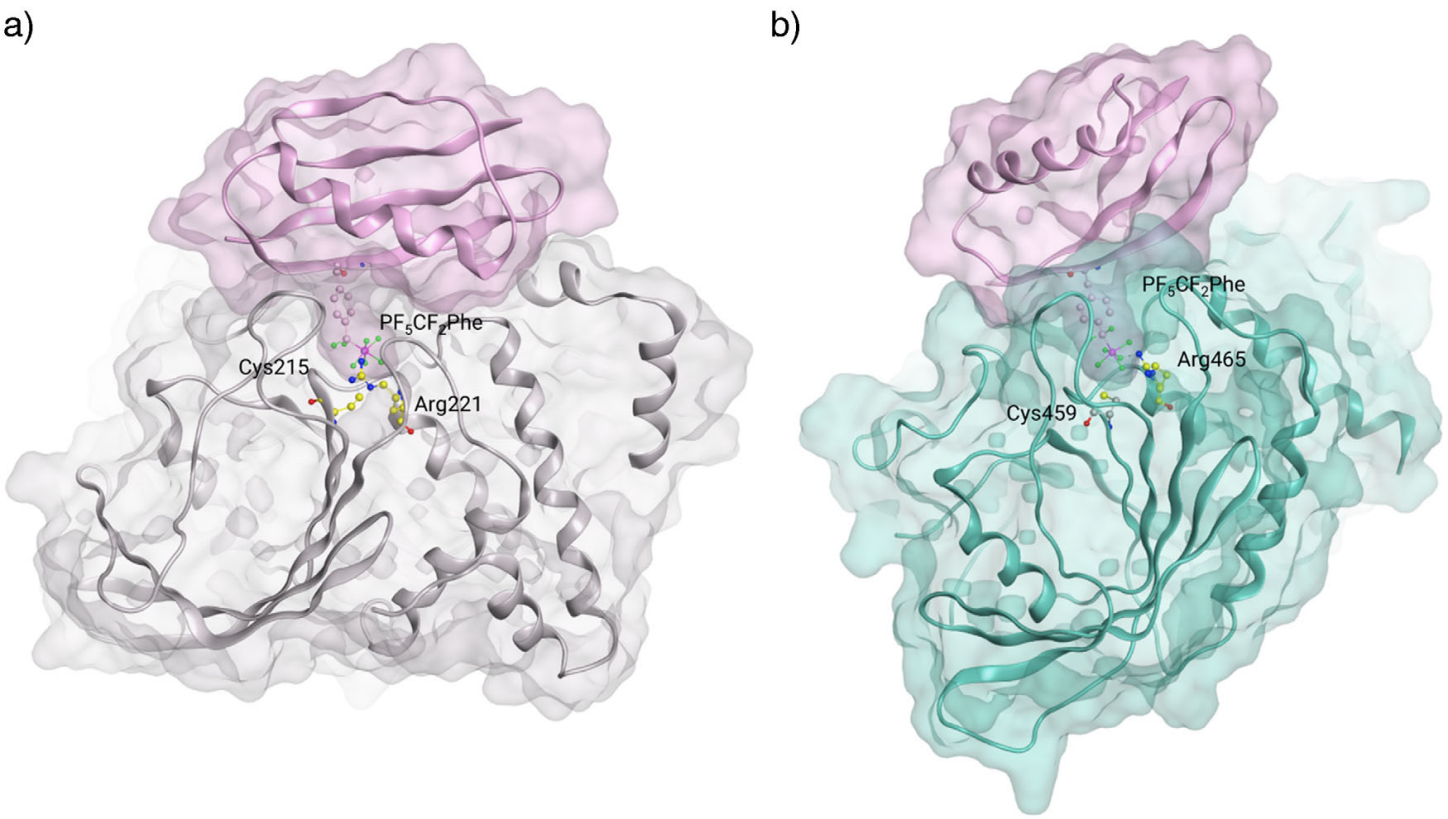
Protein–protein docking of PF_5_‐GB1 to the catalytic domains of PTP1B and SHP2. a) PF_5_CF_2_Phe of PF_5_‐GB1 reaches into the small binding cleft of PTP1B and interacts with Arg221, anchoring the protein in the binding site of the phosphatase; b) PF5‐GB1 inserts into the binding pocket of SHP2, with PF_5_CF_2_Phe extending deeply into the catalytic center, forming a salt bridge with Arg465.

In protein‐protein docking of Ubq with PTP1B, out of 100 poses generated, none was found placing the small protein in proximity to the active site of the phosphatase, which could be a reason for the missing activity. Docking of native Ubq with SHP2, however, revealed several poses with solvent‐exposed glutamic acid residues Glu24, inserting into the catalytic center. Considering the similar biological activity of the different Ubq variants (Ubq, PF5‐Ubq, PO_3_‐Ubq), it seems most likely that an invariant glutamic acid residue is responsible for the observed effect. Notably, the binding of Glu24 showed one of the highest scores and was located within a distance range to Arg465 that suggests the formation of a salt bridge. In that position, the mutation site Glu18 was found to stick out to the solvent, without forming any interactions with the active site (Figure S20).

In summary, we developed genetic code expansion tools for the site‐specific installation of the potent phosphotyrosine mimetic PF_5_CF_2_Phe in proteins. The unnatural amino acid was incorporated into three different proteins via the use of modified aminoacyl‐tRNA synthetases. PF_5_CF_2_Phe was readily taken up by cells, which is a major advantage in comparison to several other pTyr analogs, including PO_3_CF_2_Phe. Moreover, the PF_5_CF_2_ group was successfully hydrolyzed to the PO_3_CF_2_ group, showing the first example of a protein carrying this important pTyr mimetic.

PF_5_‐GB1 carrying PF_5_CF_2_Phe was a potent binder of the phosphatases PTP1B and SHP2, thereby constituting a functional phosphoprotein mimetic. The established methods are general and were demonstrated with several proteins. Thus, our approach could be applied to other proteins, yielding potential phosphoprotein mimetics, which can be used as powerful tools to investigate structural and functional effects of phosphorylation states. To encourage the adoption of this technology, the requisite plasmid pRSF‐G1PFRS has been deposited at the Addgene plasmid repository (Watertown, MA, USA).

## Supporting Information

The authors have cited additional references within the Supporting Information.^[30–51]^ The data that support the findings of this study are available in the supplementary material of this article.

## Conflict of Interests

The authors declare no conflict of interest.

## Supporting information



Supporting Information

## Data Availability

The data that support the findings of this study are available in the Supporting Information of this article.
