# Supplementary material for: Genetic Encoding of Pentafluorophosphato‐Phenylalanine Provides PF5‐Proteins as Phosphoprotein Mimetics
Source: Angew Chem Int Ed Engl. 2025 Nov 14;65(1):e18789. doi: 10.1002/anie.202518789 (PMC12759244; doi:10.1002/anie.202518789)
Supplement: Supplementary file 1 — Supporting Information [file ANIE-65-e18789-s001.docx]

**Supporting Information**

Genetic Encoding of Pentafluorophosphato-Phenylalanine Provides PF_5_-Proteins as Phosphoprotein Mimetics

Anna Magdalena Ambros, Haocheng Qianzhu, Markus Tiemann, Edan Habel, Katrin Denzinger, Hana Zupan, Matteo Accorsi, Christoph Arkona, Bettina G. Keller, Gerhard Wolber, Thomas Huber*, Jörg Rademann*

**Table of Contents**

General Methods……………………………………………………………………………………………………………...2

Chemical synthesis…………………………………………………………………………………………………………...3

Density functional theory calculations………………………………………………………………………………………6

Screening for tRNA synthetases, protein expression, and biological testing……………………………….………...10

Molecular modeling………………………………………………………………………………………………………….29

NMR spectra…………………………………………………………………………………………………………………33

Supplementary references………………………………………………………………………………………………….43

**General methods**

All chemicals used for synthesis were purchased from Sigma (Merck), ABCR, and Roth and were used without further purification. Solvents were obtained from VWR chemicals and Fisher scientific chemicals. Dry solvents were obtained from a column-based solvent purification system (MBraun, MB-SPS-800).

Moisture sensitive reactions were carried out using Schlenk technology in glassware that was heat-dried in vacuum and flushed with nitrogen.

Volatile solvents were removed by rotary evaporators from the brand Heidolph using a water bath of 40°C to 53°C. High vacuum of up to 10^-3^ mbar was generated by an oil pump by Vacuubrand. For lyophilization of aqueous samples frozen in liquid nitrogen Christ Alpha 2-4 LD plus was used.

For MPLC purification the Biotage Isolera One flash chromatography system was used with Biotage Sfär Silica HC D and Biotage Sfär C18 D cartridges. For normal phase chromatography, hexane/ethyl acetate was used as mobile phase, for reversed phase 10 mM aqueous NH_4_HCO_3_ and acetonitrile was used.

For HPLC-MS measurements the Agilent UPLC-System 1290 Infinity II was used. Samples were run on a Zorbax C18 RRHT (2.1 x 50 mm, 1.8 μm, 80 Å) column, coupled with an ESI single quadrupole mass spectrometer LC/MSD (Model# G6125C, Serial# SG2236N102) from Agilent. The mobile phase, consisting of water with 0.1% formic acid (A) and acetonitrile with 0.1 % formic acid (B), was run as a gradient starting from 5% B to 95% B.

High resolution mass spectra were measured with an analytical HPLC system (Agilent Technologies, Infinity II 1290), Zorbax Eclipse plus C18 RRHD (2.1 x 50 mm, 1.8 μM, 95 Å) column, coupled with an ESI-Q-TOF iFunnel mass spectrometer (Agilent Technologies, 6550).

^1^H-, ^13^C- and ^19^F-NMR and ^31^P-spectra of synthesized molecules were measured on JEOL ECZLS400 and JEOL ECZ600 spectrometers. Chemical shifts (δ) were referenced to the solvent peaks and are given in ppm, coupling constants (*J*) in Hz.

**Chemical synthesis**

**Sodium 4-(pentafluorophosphato-difluoromethyl)-L-phenylalanine 3**

Compound **3** was prepared from structure **6** as previously published. Briefly, the methyl ester was deprotected using a protease from *B. licheniformis* (96% yield) after which the Fmoc-group was cleaved using 20% piperidine in acetonitrile (97% yield).^[19]^ The counter ion of the PF_5_-group was exchanged using an Amberlite IRC120 Na^+^ form as previously published.^[19]^

**^1^H NMR** (500 MHz, D_2_O) δ 7.48 (d, *J* = 7.0 Hz, 2H, Ar-H), 7.33 (d, *J* = 8.1 Hz, 2H, Ar-H), 3.75 (s, 1H, CH_α_), 3.19 (dd, *J* = 18.3, 6.4 Hz, 1H, CH_2α_Phe), 2.97 (dd, *J* = 13.6, 8.4 Hz, 1H, CH_2β_Phe).

**^19^F NMR** (376 MHz, D_2_O) δ -68.56 (dp, *J* = 692.5, 43.3 Hz, F_ax_), -72.27 (ddt, *J* = 864.1, 42.9, 8.6 Hz, F_eq_), -98.16 (dt, *J* = 127.1, 8.3 Hz, CF_2_).

**HRMS (ESI)**: [M]^-^ calculated for C_10_H_10_F_7_NO_2_P^-^: 340.0343 Da, found: 340.0345 m/z

***O*-Methyl-N-(fluorenyl-9H-methoxy-carbonyl)-4-(diethoxyphosphoryl-difluoromethyl)-L-phenylalanine ester 5**

Compound **4** was prepared according to the literature. All analytical data were in accordance with the literature.^[19]^

**Ammonium *O-*methyl-N-(fluorenyl-9H-methoxy-carbonyl)-4-(pentafluorophosphato-difluoromethyl)-L-phenylalanine ester 6**

Phosphonate ester **5** (256 mg, 0.44 mmol, 1 eq.) was placed in a PFA (perfluoralkoxy polymer) flask equipped with a PTFE (polytetrafluorethylene polymer) plug and cooled to 0°C. Olah’s reagent (70:30 HF:pyridine) (623 μL, 21.81 mmol, 50 eq) and DAST (288 μL, 2.18 mmol, 5 eq.) were added slowly. The dark red reaction mixture was stirred with a magnetic stir bar and heated to 50 °C. Samples of the reaction mixture (1 µL) were diluted in acetonitrile-water (1:1, 499 µL) and analyzed by HPLC-MS. After 7 h, starting material **5** was fully consumed. The reaction mixture was cooled with an ice bath and quenched by adding dropwise TMSOMe until the pH reached 7-8. All volatiles were evaporated, and the remaining brown oil was purified by MPLC (RP-C18, 10 mM NH_4_HCO_3_/acetonitrile). 136 mg of the ammonium salt of the compound were obtained as white solid (yield = 55 %).

**[α]_D_^20^** = - 8.0 ° (c = 1, MeOH)

**^1^H NMR** (400 MHz, DMSO-*D*_6_) δ 7.88 (d, *J* = 7.8 Hz, 2H, Ar-H), 7.66 (dd, *J* = 11.9, 7.5 Hz, 2H, Ar-H), 7.41 (td, *J* = 8.0, 3.8 Hz, 2H, Ar-H), 7.35 – 7.27 (m, 2H, Ar-H), 7.25 (d, *J* = 8.0 Hz, 2H, Ar-H), 7.18 (d, *J* = 8.2 Hz, 2H, Ar-H), 4.31 – 4.15 (m, 4H, CHN 1H, Fmoc CH_2_ 2H, Fmoc CH 1H), 3.60 (s, 3H, OMe), 3.04 (dd, *J* = 13.8, 5.0 Hz, 1H, CH_2α_Phe), 2.90 (dd, *J* = 13.3, 10.7 Hz, 1H, CH_2β_Phe).

**^13^C NMR** (100 MHz, DMSO-*D*_6_) δ 172.19 (C=O methyl ester), 155.74 (C=O Fmoc), 143.62, 143.52, 142.38, 140.52, 140.51, 139.23, 137.24, 128.76, 127.69, 127.51, 127.44, 127.12, 126.88, 125.14 – 124.96 (m, CF_2_), 121.22, 119.92, 119.87, 109.63 (18 x Ar-C), 65.46 (CH_2_ Fmoc), 55.22 (CNH), 51.73 (CH_3_ methyl ester), 46.35 (CH Fmoc), 35.80 (CH_2α_).

**^19^F NMR** (376 MHz, DMSO-D6) δ -67.74 (dp, *J* = 697.0, 46.8, 44.8 Hz, F_ax_), -69.97 (ddt, *J* = 858.8, 45.2, 8.7 Hz, F_eq_), -96.99 (dp, *J* = 119.7, 8.2 Hz, CF_2_).

**^31^P NMR** (162 MHz, DMSO-D6) δ -144.23 (pdt, *J* = 858.4, 700.0, 119.9 Hz).

**HRMS (ESI)**: [M]^-^ calculated for C_26_H_22_F_7_NO_4_P^-^: 576.1175 Da, found: 576.1180 m/z

**Ammonium *O*-methyl N-(fluorenyl-9H-methoxy-carbonyl)-4-(pentafluorophosphato-carbonyl)-L-phenylalanine ester 7**

Phosphonate ester **5** (189 mg, 0.32 mmol, 1 eq.) was placed in a PFA flask equipped with a PTFE plug and cooled to 0°C. Olah’s reagent (70:30 HF:pyridine) (460 μL, 16.10 mmol, 50 eq) was added slowly. The yellow reaction mixture was stirred with a magnetic stir bar and heated to 50 °C. Samples of the reaction mixture (1 µL) were diluted in acetonitrile-water (1:1, 499 µL) and analyzed by HPLC-MS. After 4 h, starting material **5** was fully consumed and previously formed structure **6** fully hydrolyzed to **7**. The reaction mixture was cooled with an ice bath and quenched by adding dropwise TMSOMe until the pH reached 7-8. All volatiles were evaporated and the crude brown oil purified by MPLC (RP-C18, 10 mM NH_4_HCO_3_/acetonitrile). 134 mg of the ammonium salt of the compound were obtained as white solid (yield = 75 %).

**[α]_D_^20^** = - 1.4 ° (c = 5.23, MeOH)

**^1^H NMR** (600 MHz, DMSO-D6) δ 7.93 – 7.82 (m, 4H, Ar-H), 7.64 (dd, *J* = 16.4, 7.4 Hz, 2H, Ar-H), 7.45 – 7.35 (m, 2H, Ar-H), 7.33 – 7.27 (m, 2H, Ar-H), 7.24 (d, *J* = 6.7 Hz, 2H, Ar-H), 4.31 – 4.22 (m, 3H, CHN 1H, Fmoc CH_2_, 2H), 4.19 (t, *J* = 7.1 Hz, 1H, Fmoc CH), 3.62 (s, 3H, OMe), 3.07 (dd, *J* = 13.8, 4.9 Hz, 1H, CH_2α_Phe), 2.93 (dd, *J* = 13.9, 10.2 Hz, 1H, CH_2β_Phe).

**^13^C NMR** (151 MHz, DMSO-D6) δ 172.21 (C=O methyl ester), 155.89 ((C=O Fmoc), 143.75, 140.71, 129.24, 128.33, 127.63, 127.06, 125.17, 120.09 (18 x Ar-C), 65.61 (CH_2_ Fmoc), 55.18 (CNH), 51.97 (CH_3_ methyl ester), 46.56 (CH Fmoc), 36.13 (CH_2α_).

**^19^F NMR** (376 MHz, DMSO-D6) δ -61.80 (d, *J* = 45.9 Hz), -64.17 (d, *J* = 45.7 Hz), -65.04 (p, *J* = 44.5 Hz), -66.94 (p, *J* = 44.5 Hz).

**^31^P NMR** (162 MHz, DMSO-D6) δ -145.95 (pd, *J* = 891.3, 720.5 Hz).

**HRMS (ESI)**: [M]^-^ calculated for C_26_H_22_F_5_NO_5_P^-^: 554.1156 Da, found: 554.1162 m/z

**O-methyl N-(fluorenyl-9H-methoxy-carbonyl)-4-iodo-L-phenylalanine ester 8**

Structure **8** was prepared according to the literature. All analytical data were in accordance with the literature.^[19]^

**Density functional theory calculations**

Structures **Ac-1-NH_2_**, **Ac-2-NH_2_**, and **Ac-3-NH_2_** were built in pyMOL.^[30]^ The C-termini of the amino acids were capped with the NH_2_ group and the N-termini with the N-acetyl group to mimic integration of the amino acids bound in a protein. First, the search for a global minimum was performed using the GOAT algorithm^[31]^ in ORCA that relies on the semiempirical extended tight-binding (XTB) level of theory. The minimum structure from the first step was further optimized at def2-TZVP/PBE0-D3 level. Finally, the electrostatic potential surface was created using ORCA’s utility programs orca plot and orca vpot and plotted in pyMOL. An implicit solvent, either water or benzene, was used in both steps of the minima search; for XTB calculations, the analytical linearized Poisson-Boltzmann (ALPB) model^[32]^ was used and for DFT calculations the solvation model based on density (SMD).^[33]^ All DFT calculations were performed using ORCA 6.0.1^[34]^, the XTB calculations were also accessed through ORCA but rely on the implementation of XTB 6.4.0.^[35]^ Van der Waals surface areas were calculated using Molden 7.3.^[36]^

**
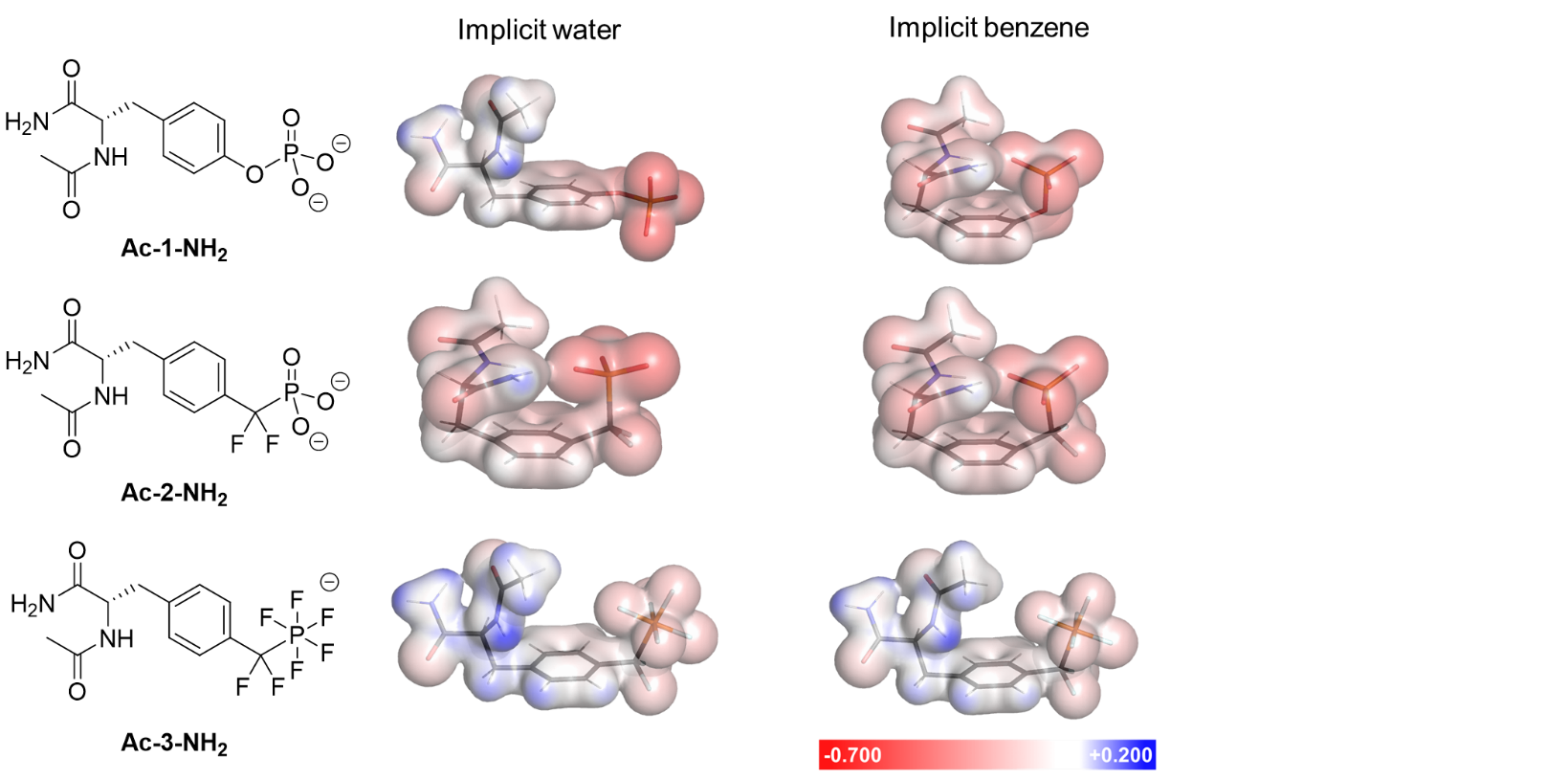
**

**Figure S1.** Electrostatic potential surfaces of N-acetylated amides of amino acids **1**-**3** in implicit water and benzene as solvents. While the energy level of all molecules is lower in water, the energy difference between water and the benzene is much higher for phosphonates **Ac-1-NH_2_** and **Ac-2-NH_2_** compared to the corresponding PF_5_-molecule **Ac-3-NH_2_** indicating the amphiphilic character of **3** (**Figure 1**).

**
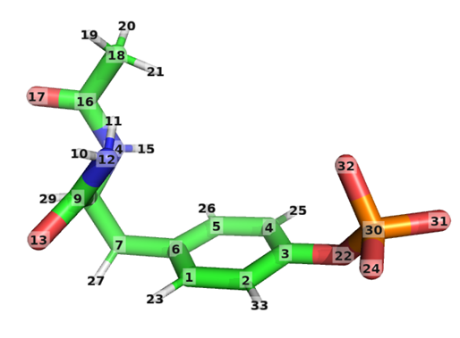
**Mulliken atomic charges and van der Waals surface area of molecule **Ac-1-NH_2_** in water

**Figure S2.** Energetically minimized 3D structure of **Ac-1-NH_2_** in water with numbered atoms.

**Table S1.** Mulliken charges and van der Waals surface area of individual atoms (**Figure S2**) of structure **Ac-1-NH_2_** in water. The total sum of atomic charges is -2.0 and the total van der Waals surface area is 325.25 Å^2^.

| Atom | | Charge | Area (Å^2^) |  | Atom | | Charge | Area (Å^2^) |  | Atom | | Charge | Area (Å^2^) |
| --- | --- | --- | --- | --- | --- | --- | --- | --- | --- | --- | --- | --- | --- |
| **1** | C | -0.294198 | 7.22 |  | **14** | N | -0.266208 | 8.80 |  | **27** | H | 0.144450 | 10.19 |
| **2** | C | -0.221500 | 7.93 |  | **15** | H | 0.270498 | 3.32 |  | **28** | H | 0.145022 | 11.04 |
| **3** | C | 0.280205 | 4.47 |  | **16** | C | 0.361146 | 4.48 |  | **29** | H | 0.153022 | 10.37 |
| **4** | C | -0.190002 | 8.88 |  | **17** | O | -0.559939 | 20.10 |  | **30** | P | 0.838623 | 28.03 |
| **5** | C | -0.333553 | 7.72 |  | **18** | C | -0.394756 | 5.74 |  | **31** | O | -0.834950 | 16.18 |
| **6** | C | 0.201222 | 4.15 |  | **19** | H | 0.141482 | 12.41 |  | **32** | O | -0.790557 | 16.31 |
| **7** | C | -0.223929 | 2.26 |  | **20** | H | 0.165375 | 12.72 |  | **33** | H | 0.142585 | 10.18 |
| **8** | C | -0.001970 | 0.46 |  | **21** | H | 0.148605 | 10.62 |  |  |  |  |  |
| **9** | C | 0.260364 | 3.63 |  | **22** | O | -0.424490 | 6.75 |  |  |  |  |  |
| **10** | H | 0.283320 | 4.30 |  | **23** | H | 0.148316 | 10.37 |  |  |  |  |  |
| **11** | H | 0.288338 | 0.08 |  | **24** | O | -0.820618 | 14.44 |  |  |  |  |  |
| **12** | N | -0.404206 | 18.11 |  | **25** | H | 0.132858 | 12.53 |  |  |  |  |  |
| **13** | O | -0.508226 | 20.44 |  | **26** | H | 0.163672 | 11.03 |  |  |  |  |  |

Mulliken atomic charges and van der Waals surface area of molecule **Ac-2-NH_2_** in water


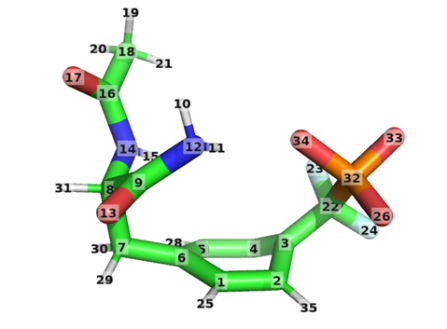


**Figure S3.** Energetically minimized 3D structure of **Ac-2-NH_2_** in water with numbered atoms.

**Table S2.** Mulliken charges and van der Walls surface area of individual atoms (**Figure S3**) of structure **Ac-2-NH_2_** in water. The total sum of atomic charges is -2.0 and the total surface area is 344.07 Å^2^.

| Atom | | Charge | Area (Å^2^) |  | Atom | | Charge | Area (Å^2^) |  | Atom | | Charge | Area (Å^2^) |
| --- | --- | --- | --- | --- | --- | --- | --- | --- | --- | --- | --- | --- | --- |
| **1** | C | -0.226002 | 7.18 |  | **14** | N | -0.254893 | 8.33 |  | **27** | H | 0.148796 | 12.84 |
| **2** | C | -0.275234 | 5.96 |  | **15** | H | 0.248292 | 3.62 |  | **28** | H | 0.147828 | 12.33 |
| **3** | C | 0.158927 | 2.78 |  | **16** | C | 0.322745 | 6.37 |  | **29** | H | 0.143740 | 10.24 |
| **4** | C | -0.186460 | 7.15 |  | **17** | O | -0.564647 | 19.08 |  | **30** | H | 0.143537 | 10.95 |
| **5** | C | -0.292213 | 7.97 |  | **18** | C | -0.396577 | 6.85 |  | **31** | H | 0.133987 | 10.31 |
| **6** | C | 0.085716 | 3.96 |  | **19** | H | 0.164523 | 12.37 |  | **32** | P | 0.728763 | 21.80 |
| **7** | C | -0.124619 | 2.31 |  | **20** | H | 0.151839 | 12.71 |  | **33** | O | -0.799230 | 16.00 |
| **8** | C | 0.056770 | 0.45 |  | **21** | H | 0.129495 | 12.26 |  | **34** | O | -0.750784 | 10.86 |
| **9** | C | 0.198637 | 3.56 |  | **22** | C | 0.233179 | 0.23 |  |  |  |  |  |
| **10** | H | 0.268284 | 4.30 |  | **23** | F | -0.231261 | 16.57 |  |  |  |  |  |
| **11** | H | 0.282987 | 2.13 |  | **24** | F | -0.229100 | 18.33 |  |  |  |  |  |
| **12** | N | -0.376061 | 15.20 |  | **25** | H | 0.154592 | 11.91 |  |  |  |  |  |
| **13** | O | -0.523600 | 21.14 |  | **26** | O | -0.828658 | 14.24 |  |  |  |  |  |

Mulliken atomic charges and van der Waals surface area of molecule **Ac-3-NH_2_** in water


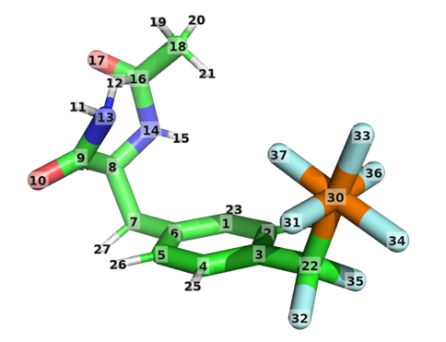


**Figure S4.** Energetically minimized 3D structure of **Ac-3-NH_2_** in water with numbered atoms.

**Table S3.** Mulliken charges and van der Waals surface area of individual atoms (**Figure S4**) of structure **Ac-2-NH_2_** in water. The total sum of atomic charges is -2.0 and the total surface area is 349.87 Å^2^.

| Atom | | Charge | Area (Å^2^) |  | Atom | | Charge | Area (Å^2^) |  | Atom | | Charge | Area (Å^2^) |
| --- | --- | --- | --- | --- | --- | --- | --- | --- | --- | --- | --- | --- | --- |
| **1** | C | -0.275482 | 7.81 |  | **14** | N | -0.273048 | 8.66 |  | **27** | H | 0.148659 | 10.38 |
| **2** | C | -0.248158 | 7.85 |  | **15** | H | 0.279213 | 3.98 |  | **28** | H | 0.149353 | 11.10 |
| **3** | C | 0.196076 | 3.02 |  | **16** | C | 0.367852 | 3.87 |  | **29** | H | 0.158364 | 11.60 |
| **4** | C | -0.234229 | 7.67 |  | **17** | O | -0.559711 | 18.98 |  | **30** | P | 0.919970 | 10.77 |
| **5** | C | -0.270517 | 7.23 |  | **18** | C | -0.408450 | 5.80 |  | **31** | F | -0.354347 | 14.16 |
| **6** | C | 0.200499 | 4.06 |  | **19** | H | 0.154621 | 11.35 |  | **32** | F | -0.202807 | 15.60 |
| **7** | C | -0.232075 | 2.26 |  | **20** | H | 0.166829 | 12.72 |  | **33** | F | -0.360487 | 15.01 |
| **8** | C | 0.000737 | 0.49 |  | **21** | H | 0.150989 | 11.84 |  | **34** | F | -0.342115 | 14.60 |
| **9** | C | 0.258385 | 3.12 |  | **22** | C | 0.230391 | 0.31 |  | **35** | F | -0.201728 | 15.48 |
| **10** | O | -0.507070 | 20.61 |  | **23** | H | 0.155089 | 11.19 |  | **36** | F | -0.355250 | 14.06 |
| **11** | H | 0.283310 | 4.30 |  | **24** | H | 0.159060 | 10.80 |  | **37** | F | -0.355023 | 13.41 |
| **12** | H | 0.289998 | 0.11 |  | **25** | H | 0.157309 | 10.94 |  |  | | | |
| **13** | N | -0.403748 | 14.35 |  | **26** | H | 0.157542 | 10.41 |  |  |  |  |  |

**Screening for tRNA synthetases, protein expression, and biological testing**

**Screening for functional *Mj*TyrRS enzymes recognizing PF_5_CF_2_Phe**

To carry out selection based on the *Mj*Tyr OTS, the previously constructed library plasmid pBK-*Mj*TyrRS^[21]^ as transformed into *E. coli* DH10B cells harboring the selection plasmid pBAD-H6RFP^[21]^. The recovery culture after transformation was directly inoculated into two flasks each with 25 mL LB medium containing 100 mg/L carbenicillin and 50 mg/L kanamycin, supplied with 0.2% arabinose. Each “+” sample contained 1 mM PF_5_CF_2_Phe, while the “–” samples were not provided with ncAA. Overnight expression at 37^o^C led to a well-detectable level of red fluorescence. Cells pelleted from 400 μL of overnight culture were resuspended in 8 mL PBS buffer (137 mM NaCl, 2.7 mM KCl, 10 mM Na_2_HPO_4_, 1.8 mM KH_2_PO_4_, pH 7.4), yielding a concentration suitable for cell sorting by FACS on an Aria Fusion high speed cell sorter (BD Biosciences). General gating was based on monitoring forward and side scattering to select the expected size of bacteria. Cells were sorted according to the level of RFP expression in each selection round. Positive selection rounds identified those RS mutants in the library, which were capable of reading through the amber codon. Negative selection rounds identified RS mutants unable to support amber codon read-through without the addition of ncAA. Sorted cells from each round were directly inoculated into another 25 mL fresh LB medium containing 100 mg/L carbenicillin, 50 mg/L kanamycin and 0.2% arabinose, with or without ncAA, to iteratively repeat the overnight expression.

Three rounds of selection were carried out on the *Mj*TyrRS library (**Figure S5**). The top 9.3% of cells with high RFP levels were selected from the **1P+** sample (as indicated by shadow and arrow in **Figure S5**), collecting 2.0 × 10^6^ cells. The collected cells were subjected to the following round of negative selection (**2N–**), from where cells with low RFP expression levels (52.1% of the total) were collected (2.0 × 10^6^ cells). These cells were aliquoted to inoculate media with positive (**3P+**) and negative (**3P–**) conditions. These two samples displayed a dramatic difference in fluorescence, with 56.6% of the cells displaying high RFP levels in the 3P+ sample compared to 1.7% in the **3P–** sample using the same gating conditions. Cells with high RFP levels in both the “total positive” and the top 5% fraction were collected respectively from the **3P+** sample (1.0×10^5^ cells were kept for storage). Aliquots with 2,000 cells each were recovered on LB agar plates containing 100 mg/L carbenicillin and 50 mg/L kanamycin. 60 individual colonies from each fraction were inoculated and analyzed in 96-well plates under positive (+1 mM PF_5_CF_2_Phe) and negative (–ncAA) growth conditions. The fluorescence level was measured after overnight incubation at 37 ^o^C using a TECAN Infinite 200 Pro M Plex plate reader (Tecan, Switzerland). The RFP read-outs were normalized by the OD_600_ of the cell culture (**Figure S7**). The behaviour of the candidates was scored by their corresponding ratio of RFP+/RFP–. Seven candidates with the highest RFP level and best specificity were chosen for sequencing. Every one of them displayed different mutation sets (**Table S4**).

**Screening for functional G1PylRS enzymes recognizing PF_5_CF_2_Phe**

To carry out the selection based on the G1Pyl OTS, the previously constructed library plasmid pBK-G1RS^[37]^ as transformed into *E. coli* DH10B cells harboring the selection plasmid pBAD-H6RFP^[21]^. Following recovery from transformation, the culture was directly inoculated into a flask with 25 mL LB medium containing 100 mg/L carbenicillin and 50 mg/L kanamycin, supplied with 0.2% L-arabinose and 1 mM PF_5_CF_2_Phe, which served as the sample for the first round of positive selection (**1P+**). Overnight expression at 37 ^o^C led to a readily detectable level of RFP expression. 400 μL of cells were resuspended in 8 mL PBS buffer (137 mM NaCl, 2.7 mM KCl, 10 mM Na_2_HPO_4_, 1.8 mM KH_2_PO_4_, pH 7.4) yielding a concentration suitable for cell sorting by FACS on an Aria Fusion high speed cell sorter (BD Biosciences, USA; **Figure S6**).

Cells with high RFP levels were selected from the **1P+** sample (0.4% of the total population), collecting 2.0×10^5^ cells. The cells collected were subjected to a following round of negative selection without the addition of PF_5_CF_2_Phe and regrown as sample **2N–**, from where cells with low RFP expression levels (66.3%) were collected (8.0×10^5^ cells). These cells were aliquoted to inoculate media with positive (**3P+**) and negative (**3P–**) conditions. The RFP-positive cells (1.9%) from the **3P+** sample were collected (2.0×10^5^ cells) and recovered under negative condition to obtain **4N–**. Following sorting (4.0×10^5^ cells), cells showing the lowest level of RFP fluorescence (58.6%) were selected from the **4N–** sample. They were aliquoted to be recovered as **5P+** and **5P–,** respectively. The cell population with high RFP fluorescence in the **5P+** sample was 44.1% with PF_5_CF_2_Phe provided compared to 14.2% without ncAA, indicating the successful accumulation of active G1PylRS variants specific for PF_5_CF_2_Phe. 2.0×10^5^ cells collected from the top 5% RFP fluorescent cells of the **5P+** sample were recovered for storage. An aliquot of 2,000 cells were allowed to recover on LB agar plates containing 100 mg/L carbenicillin and 50 mg/L kanamycin, and individual clones were analyzed using 96-well plates. 120 colonies were inoculated into both positive (with 1 mM PF_5_CF_2_Phe) and negative (without ncAA) growth conditions. The fluorescence level was measured after expression overnight, using a TECAN Infinite 200 Pro M Plex plate reader (Tecan, Switzerland) and normalized by the OD_600_ of the cell culture (**Figure S7**). 15 candidates with the best efficiency and specificity were chosen for sequencing. Eight individually different sequences were found (**Table S4**).

**Table S4.** Mutations found in selected colonies with *Mj*TyrRS or G1PylRS variants that recognize PF_5_CF_2_Phe. Information of the residues selected for randomization appeared in wild-type RS is given in grey rows. Mutation sets with blue/red/yellow/green background color are of the same variant.

| *RS Variants* | *Randomized Sites* | | | | | |  |
| --- | --- | --- | --- | --- | --- | --- | --- |
| *Mj*TyrRS-wt | Y32 | L65 | N109 | D158 | I159 | L162 |  |
| MjPF05^a^ | V | V | M | G | G | Q |  |
| MjPF16^a^ | G | G | K | G | V | A |  |
| MjPF19^a^ | A | G | Q | G | G | N |  |
| MjPF32^a^ | G | A | R | S | S | D |  |
| MjPF47^a^ | A | G | H | G | A | F |  |
| MjPF54^a^ | A | G | E | G | V | Q |  |
| MjPF60^a^ | V | G | C | G | C | I |  |
| *Mm*PylRS-wt | L305 | Y306 | N346 | V348 | Y384 | V401 | W417 |
| G1PylRS-wt | L124 | Y125 | N165 | V167 | Y204 | A221 | W237 |
| G1PF13 | F | L | A | V | W | I | R |
| G1PF15 | F | L | A | V | W | I | R |
| G1PF16^b^ | H | L | S | V | W | I | R |
| G1PF23 | H | L | S | V | F | L | R |
| G1PF28 | H | L | N | V | W | I | R |
| G1PF36 | H | L | S | V | W | I | R |
| G1PF38 | H | L | S | V | W | I | R |
| G1PF48 | H | L | S | V | W | I | R |
| G1PF55 | M | L | A | C | F | L | R |
| G1PF57 | H | L | S | A | F | I | R |
| G1PF62 | H | L | S | A | F | I | R |
| G1PF95 | M | L | A | A | W | L | R |
| G1PF103 | H | L | N | V | W | I | R |
| G1PF104 | F | L | A | C | F | V | R |
| G1PF111 | H | L | S | V | W | I | R |

^a^ RS mutants contain additional mutations F108W^[38]^and D286R^[39]^.

^b^ G1PF16 was cloned into the pRSF vector to produce the synthetase plasmid pRSF-G1PFRS for large scale expression. This plasmid has been deposited at Addgene (Watertown, MA, USA).

***In vivo* protein expression and purification**

The gene encoding G1PF16 (**Table S5**) was cloned into a high-copy number pRSF plasmid with a kanamycin resistance marker, same as our previously reported construct^[21,25,37]^ to yield pRSF-G1PFRS as the synthetase plasmid. The expression of G1PF16 is controlled by *T7* promoter, and ^G1Pyl^tRNA_CUA_ is under *lpp* promoter.

Site-specific incorporation of PF_5_CF_2_Phe was achieved in *E. coli* B95.ΔAΔ*fab* cells^[25]^ co-transformed with the synthetase plasmid and the pCDF plasmid containing the amber codon interrupted gene of the protein of interest (**Table S5**). The transformed cells were grown at 37 °C in LB medium containing 25 mg/L kanamycin and 25 mg/L spectinomycin. An aliquot (1 mL) of an overnight culture was used to inoculate 100 mL LB medium supplemented with the same antibiotic as above and 1 mM of PF_5_CF_2_Phe. The cells were grown at 37 °C to an OD_600_ of 0.6. At this point, the temperature was reduced to 25 °C and protein expression was induced by the addition of 1 mM isopropyl β-D-1-thiogalactopyranoside (IPTG).

After expression for 16 h, the cells were harvested by centrifugation. Following resuspension in buffer A (50 mM Tris-HCl pH 7.5, 300 mM NaCl, 5% glycerol, 20 mM imidazole), the cells were lyzed using an Avestin Emulsiflex C5 system (Avestin, Canada) using two passes with a pressure of 10,000–15,000 psi. The cell lysates were centrifuged for 1 h at 24,000 *g*. The supernatant was loaded onto a 1 mL His GraviTrap column (Cytiva, USA). The column was washed with 25 column volumes buffer A and the protein was eluted with 5 column volumes buffer B (same as buffer A but with 500 mM imidazole). Afterwards, the buffer was exchanged to assay buffer (PBS for RFP13-TAG; 20 mM MES, pH 6.5, and 150 mM NaCl for ZiPro) using an Amicon ultrafiltration centrifugal tube (Merck Millipore, USA) with a molecular weight cut-off of 3 kDa.

**Table S5.** DNA and corresponding amino acid sequences of the proteins used in the current study.

| Protein | DNA sequence | Amino acid sequence^a^ |
| --- | --- | --- |
| G1PF16 | ATGGTGGTGAAATTTACCGATAGCCAGATTCAGCATCTGATGGAATATGGTGATAATGATTGGAGCGAAGCCGAATTTGAAGATGCAGCAGCACGTGATAAAGAATTTAGCAGCCAGTTTAGCAAACTGAAAAGCGCCAATGATAAAGGCCTGAAAGATGTTATTGCAAATCCGCGTAATGATCTGACCGATCTGGAAAACAAAATTCGCGAAAAACTGGCAGCCCGTGGTTTTATTGAAGTTCATACCCCGATTTTTGTGAGCAAAAGCGCACTGGCAAAAATGACCATTACCGAAGATCATCCGCTGTTCAAACAGGTGTTTTGGATTGATGATAAACGTGCACTGCGTCCGATGCATGCAATGAATCATTTGAAAGTTATGCGTGAACTGCGCGATCATACCAAAGGTCCGGTTAAAATCTTTGAAATTGGTAGCTGCTTTCGCAAAGAAAGCAAAAGCAGTACCCATCTGGAAGAATTTACCATGCTGAGCCTGGTAGAAATGGGTCCTGATGGTGATCCGATGGAACATCTGAAAATGTATATTGGCGATATCATGGATGCCGTTGGTGTTGAATATACCACCAGTCGTGAAGAATCAGATGTTTGGGTTGAAACCCTGGACGTGGAAATTAATGGCACCGAAGTTGCAAGCGGTATTGTTGGTCCGCATAAACTGGATCCGGCACATGATGTGCATGAACCGAGGGCAGGTATTGGTTTTGGTCTGGAACGTCTGCTGATGCTGAAAAATGGTAAAAGCAATGCACGCAAAACCGGCAAAAGTATTACCTATCTGAATGGCTACAAACTGGATTAA | MVVKFTDSQIQHLMEYGDNDWSEAEFEDAAARDKEFSSQFSKLKSANDKGLKDVIANPRNDLTDLENKIREKLAARGFIEVHTPIFVSKSALAKMTITEDHPLFKQVFWIDDKRALRPMHAMNHLKVMRELRDHTKGPVKIFEIGSCFRKESKSSTHLEEFTMLSLVEMGPDGDPMEHLKMYIGDIMDAVGVEYTTSREESDVWVETLDVEINGTEVASGIVGPHKLDPAHDVHEPRAGIGFGLERLLMLKNGKSNARKTGKSITYLNGYKLD |
| His_6_-TAG-mRFP1 | ATGCACCACCATCACCATCACTAGGCCAGTAGTGAAGACGTTATCAAGGAGTTTATGCGTTTCAAAGTACGTATGGAGGGTAGTGTTAACGGACACGAATTTGAGATCGAGGGAGAGGGGGAAGGTCGTCCTTACGAGGGAACTCAAACGGCCAAATTAAAGGTGACCAAAGGTGGGCCCTTGCCATTCGCGTGGGACATCTTGTCACCCCAGTTCCAGTACGGGTCGAAGGCATACGTAAAACACCCAGCGGACATTCCTGACTATCTTAAGTTATCTTTCCCGGAAGGTTTTAAATGGGAACGCGTGATGAACTTTGAGGATGGGGGGGTTGTTACGGTGACACAAGACTCCTCATTGCAAGATGGAGAGTTTATCTATAAAGTCAAACTTCGCGGCACCAATTTTCCATCTGACGGTCCTGTAATGCAGAAAAAAACAATGGGCTGGGAAGCCTCCACAGAACGTATGTACCCCGAAGATGGAGCTTTAAAGGGCGAAATTAAAATGCGCTTAAAACTTAAAGACGGCGGCCATTACGACGCCGAAGTGAAAACGACGTATATGGCTAAGAAACCCGTCCAGCTTCCGGGAGCCTATAAAACTGACATCAAACTGGATATTACATCACACAACGAAGATTATACTATTGTCGAACAGTACGAACGCGCCGAAGGCCGCCATTCAACGGGAGCATAA | MHHHHHHXASSEDVIKEFMRFKVRMEGSVNGHEFEIEGEGEGRPYEGTQTAKLKVTKGGPLPFAWDILSPQFQYGSKAYVKHPADIPDYLKLSFPEGFKWERVMNFEDGGVVTVTQDSSLQDGEFIYKVKLRGTNFPSDGPVMQKKTMGWEASTERMYPEDGALKGEIKMRLKLKDGGHYDAEVKTTYMAKKPVQLPGAYKTDIKLDITSHNEDYTIVEQYERAEGRHSTGA |
| Ubq-E18TAG | ATGCAGATCTTCGTGAAGACTCTGACTGGTAAGACCATCACCCTCGAGGTTTAGCCCAGTGACACCATTGAGAATGTCAAGGCAAAGATCCAAGATAAGGAAGGCATCCCTCCTGACCAGCAGAGGCTGATCTTTGCTGGAAAACAGCTGGAAGATGGGCGCACCCTGTCTGACTACAACATCCAGAAAGAGTCCACCCTGCACCTGGTACTCCGTCTCAGAGGTGGAAGTCATCATCATCATCACCACTAA | MQIFVKTLTGKTITLEVXPSDTIENVKAKIQDKEGIPPDQQRLIFAGKQLEDGRTLSDYNIQKESTLHLVLRLRGGSHHHHHH |
| GB1-T17TAG | atgGCTTCTATGACCGGTATGACCTACAAACTGATCCTGAACGGTAAAACCCTGAAAGGTGAAACCTAGACCGAAGCGGTTGACGCGGCGACCGCGGAAAAAGTTTTCAAACAGTACGCGAACGACAACGGTGTTGACGGTGAATGGACCTACGACGACGCGACCAAAACCTTCACCGTTACCGAAGAAAACCTGTATTTTCAGGGCCATCACCATCACCATCACTAA | MASMTGMTYKLILNGKTLKGETXTEAVDAATAEKVFKQYANDNGVDGEWTYDDATKTFTVTEENLYFQGHHHHHH |

^a^ X indicates the positions of PF_5_CF_2_Phe.


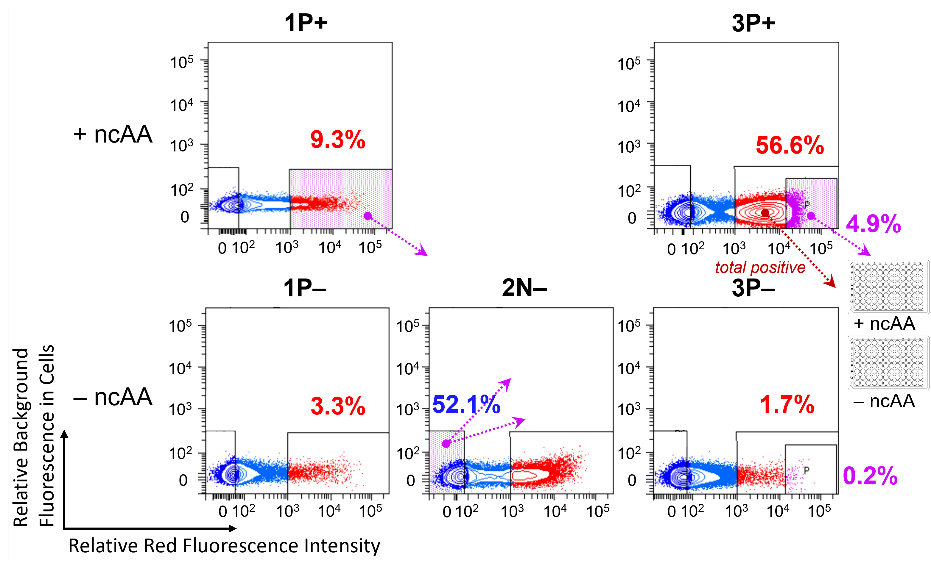


**Figure S5.** FACS experiments for selection of active and specific *Mj*TyrRS enzymes that recognize PF_5_CF_2_Phe. The horizontal axis indicates the relative intensity of red fluorescence. The x-axis indicates red fluorescence intensity (excitation at 560 nm), and the y-axis reflects background fluorescence upon excitation at 488 nm. Positive samples (**1P+**, **3P+**, **5P+**) were prepared in the presence of 1 mM PF_5_CF_2_Phe, while negative samples (**1P–**, **2N–**, **3P–**, **4N–**, **5P–**) were cultured without ncAA. Populations collected for subsequent rounds are shaded in purple, with percentages denoting the gated fluorescence-positive fraction. Arrows indicate the direction of the iterative enrichment strategy. Both the top 5% and the “total positive” fraction from the **3P+** sample were collected for further characterization.


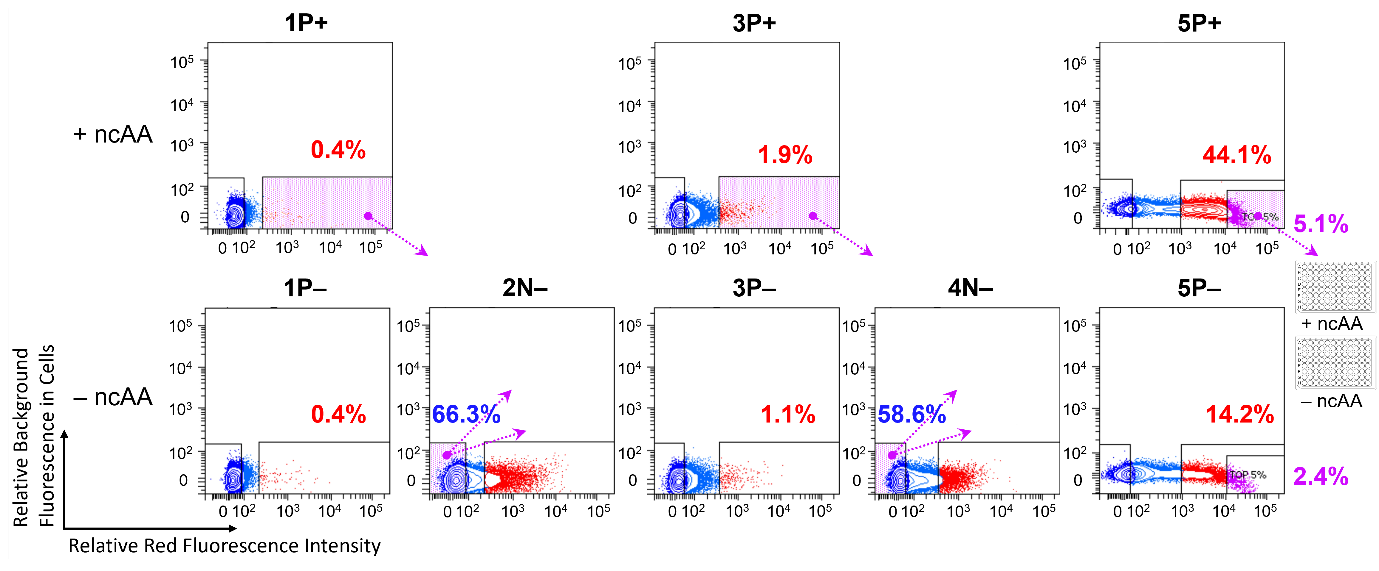


**Figure S6.** FACS experiments for selection of active and specific G1PylRS enzymes that recognize PF_5_CF_2_Phe. Annotations are the same as in **Figure S2**. The top 5% fraction from the **5P+** sample was collected for further characterization.

**A**

**
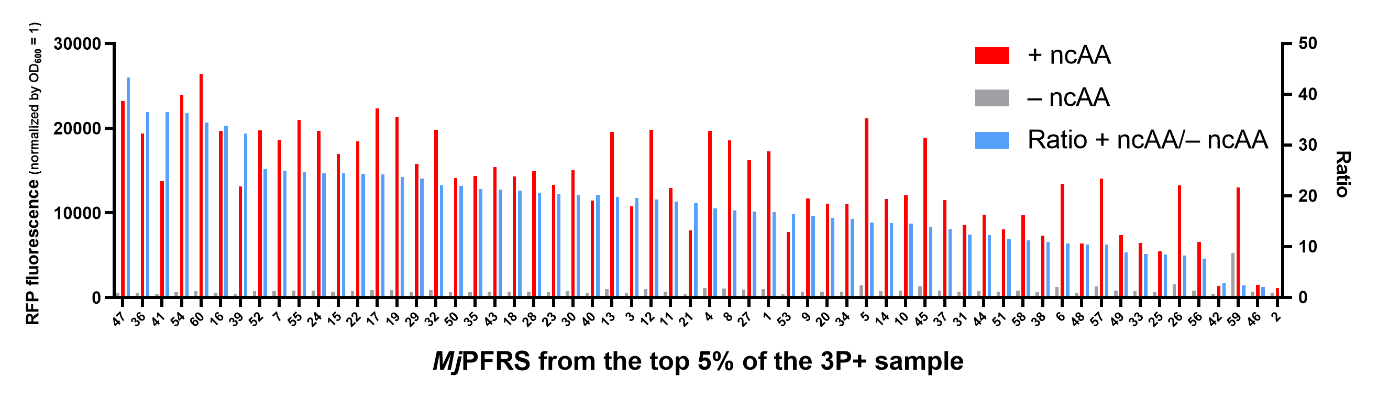
**

**B**

**
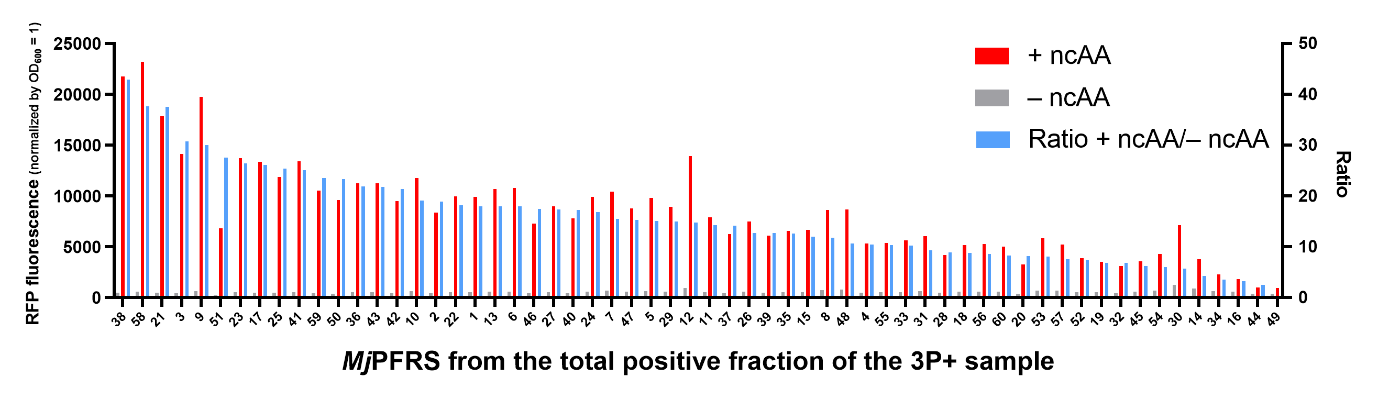
**

**Figure S7.** Activity and specificity characterization of *Mj*PFRS variants for PF_5_CF_2_Phe incorporation. In the third round of FACS selection experiment using the *Mj*TyrRS library, cells collected from the (A) top 5% /(B) total positive fraction of the 3P+ sample were cultured on 96-well plates with/without 1 mM PF_5_CF_2_Phe. Red fluorescence intensity indicative of the readthrough efficiency of the amber-interrupted reporter gene was then measured. The plot presents the colonies ranked in a descending order based on the ratio of red fluorescence in the + ncAA wells compared to the – ncAA wells. This ranking highlights the candidates with the highest activity and specificity for PF_5_CF_2_Phe incorporation.


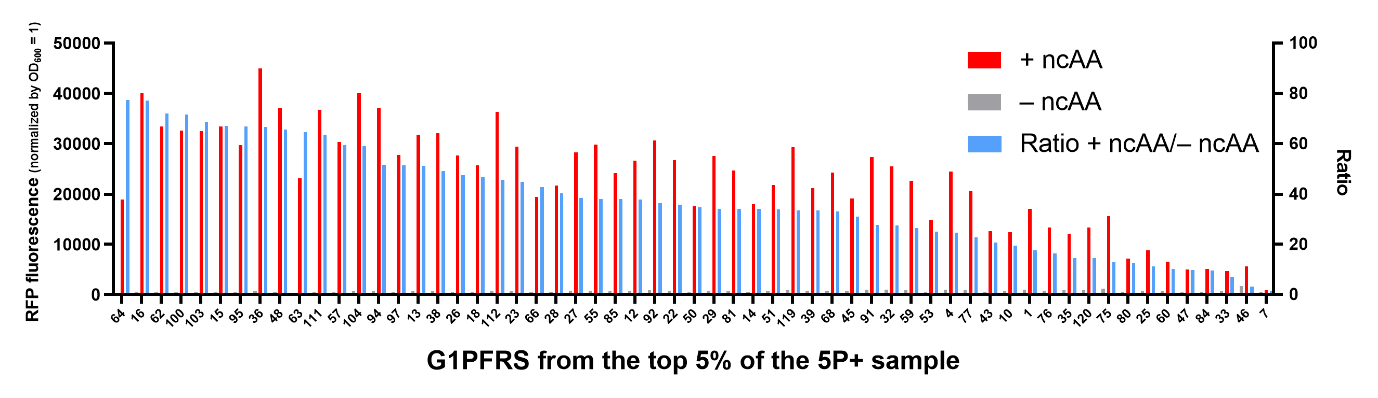


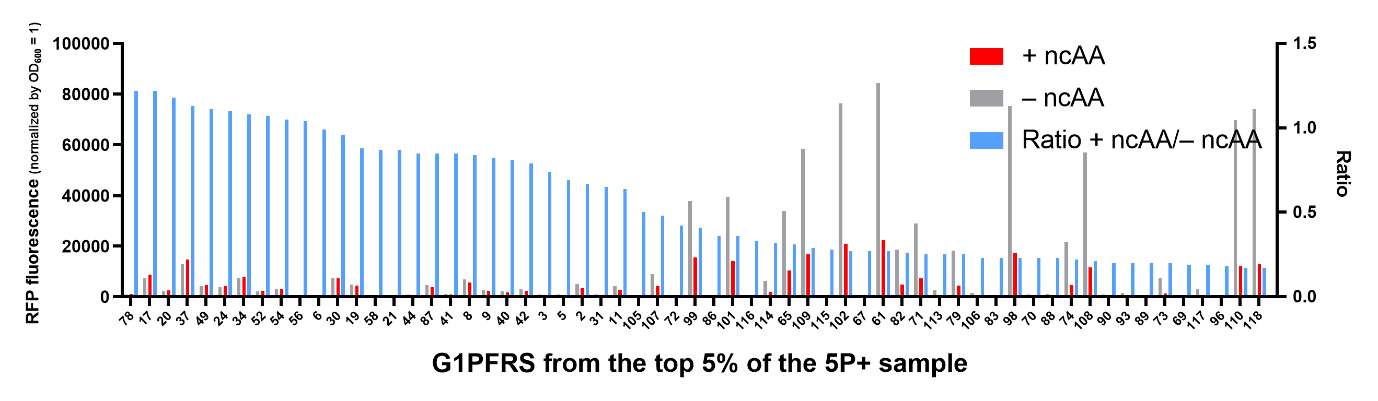


**Figure S8.** Activity and specificity characterization of G1PFRS variants for PF_5_CF_2_Phe incorporation. In the fifth round of FACS selection experiment using the G1PylRS library, cells collected from the top 5% fraction of the 5P+ sample were cultured on 96-well plates with/without 1 mM PF_5_CF_2_Phe. Expression level of the amber-interrupted RFP is measured. A total of 120 mutants were analyzed and ranked in descending order based on the ratio of red fluorescence intensity in the + ncAA condition relative to the – ncAA control.

*
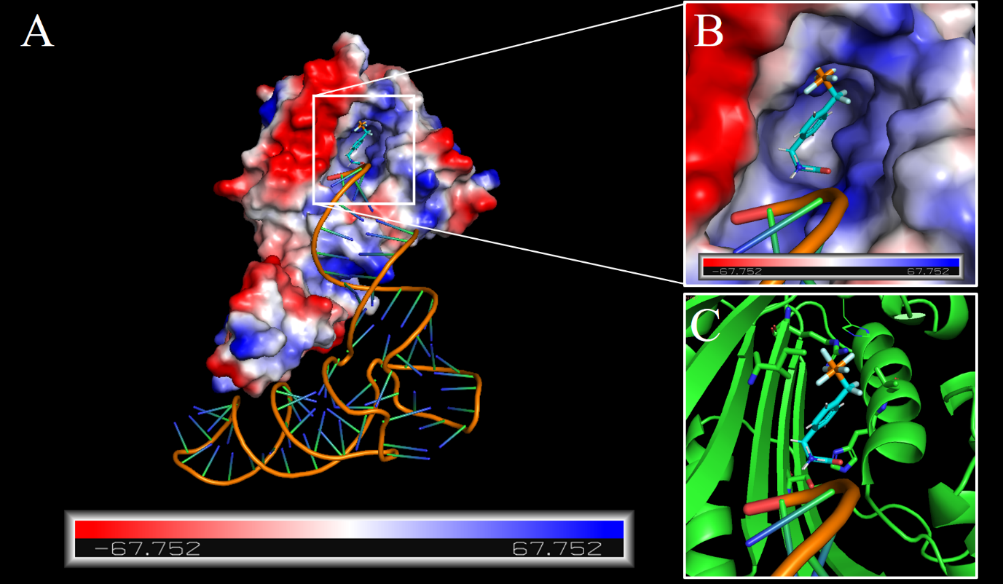
*

**Figure S9.** Computationally derived model of G1PF16 with tRNA and PF_5_CF_2_Phe bound as substrate. The residue changes relative to the wild type G1 tRNA synthetase (L124H, Y125L, N165S, Y204W, A221I, W237R) generate a deep, positively charged and hydrophobic pocket to bind the negatively charged PF_5_CF_2_Phe. A) and B) van der Waals surface with mapped vacuum electrostatics. C) Close-up of mutated residues in the amino acid binding site. Side chains of the convergent arginine mutation at position 237 and histidine at position 225 are in close proximity to the negatively charged PF_5_ group of the unnatural amino acid. The model of the G1PF16:tRNA complex was generated using AlphaFold3.^[40]^ PF5CF2Phe was modelled into the structure starting from the non-canonical amino acid (3-iodo-L-phenylalanine) ligand position in the crystal structure of M. mazei Go1 PylRS (PDB: 4TQD). Vacuum electrostatic surfaces are calculated and visualized in pyMOL.^[30]^

**
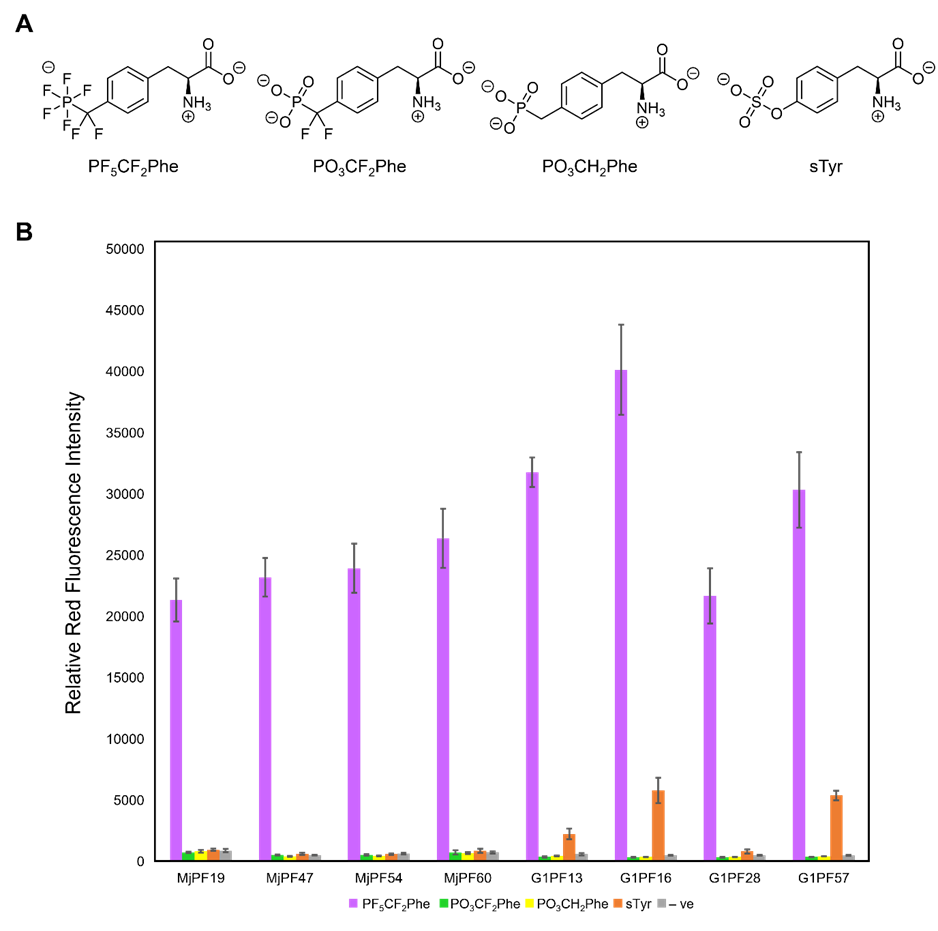
**

**Figure S10.** (A) Structure comparison of ncAAs as tyrosine derivatives with negatively charged bulky substituents in the *para* position. (B) Substrate specificity test of active PFRS mutants. Four mutants from each of the *Mj* and G1 OTS were selected. DH10B cells carrying these RS mutants co-transformed with the pBAD-H6RFP plasmid were cultured in 96-well plates. 1mM of ncAA is supplied to the corresponding wells. Error bars indicate standard deviation from three biological replicates.


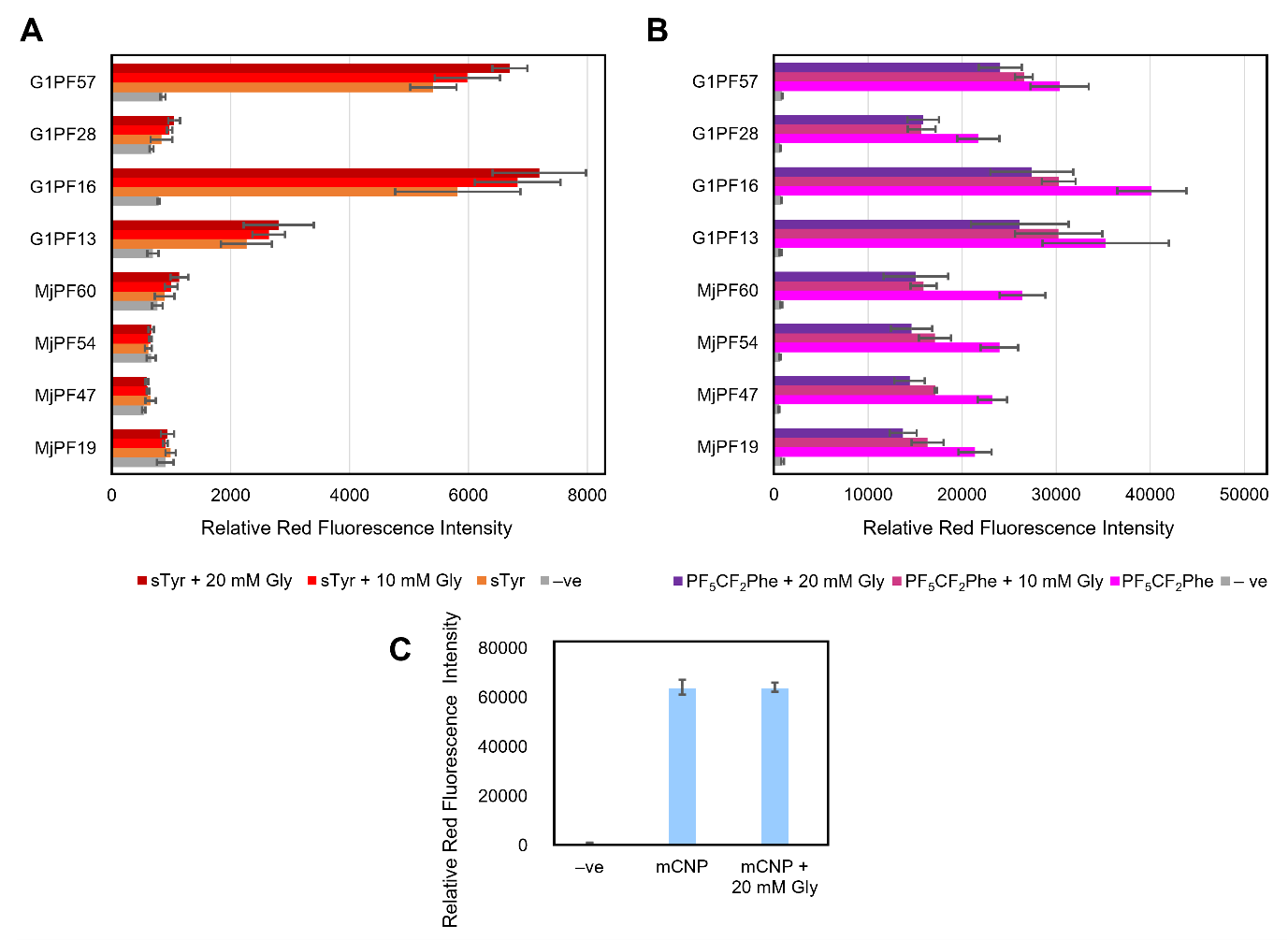


**Figure S11.** Effect of additional glycine on the cellular uptake of negatively charged ncAAs. DH10B cells carrying eight PFRS mutants co-transformed with the pBAD-H6RFP plasmid were cultured in 96-well plates. 1 mM of (A) sTyr /(B) PF_5_CF_2_Phe, in addition to 10 mM or 20 mM glycine, was supplied to the corresponding wells. (C) Control experiment was carried out using our previously reported G1PylRS mutant that incorporates *meta*-cyanopyridylalanine (mCNP).^[22]^ Upon addition of 20 mM glycine, no change was observed for the incorporation of mCNP at 1 mM in medium.

**Protein mass spectrometry**

Protein MS analysis was performed on an Orbitrap Fusion™ Tribrid™ mass spectrometer (Thermo Fisher Scientific, USA) connected to a Thermo Fisher Scientific UltiMate 3000 HPLC system equipped with ZORBAX 300SB-C3, 3.5 µm, 4.6 x 50 mm HPLC column (Agilent Technologies, USA). Approximately 50 pmol of sample was injected using a 500 µL/min linear gradient of solvent A (0.1% (v/v) formic acid in water) and solvent B (0.1% (v/v) formic acid in acetonitrile), ramping solvent B from 5% solvent B at the start to 80% after 12 min. Data were collected using an electrospray ionization (ESI) source in positive ion mode. Protein intact mass was determined by deconvolution using the program Xcalibur 3.0.63 (Thermo Fisher Scientific, USA).


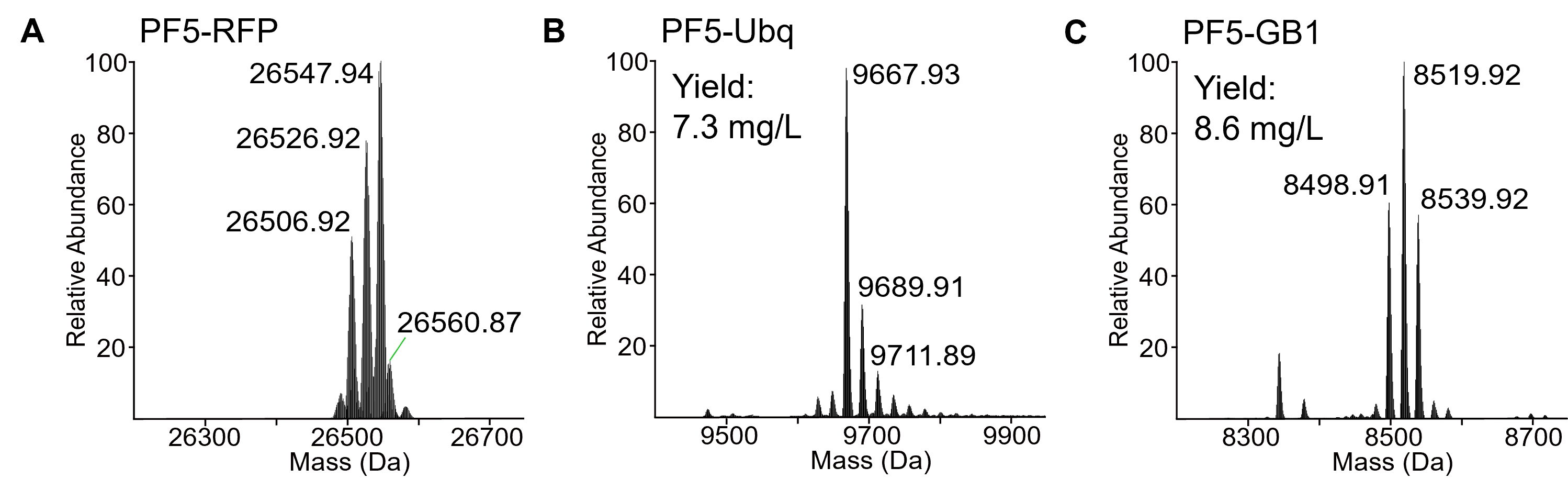


**Figure S12.** Protein mass spectrometric analysis of proteins with PF_5_CF_2_Phe incorporated. (A) The calculated mass of PF_5_-RFP (after loss of the N-terminal methionine) is 26545.71 Da. The observed peaks at 26526.92 Da and 26506.92 Da likely correspond to the loss of one and two molecules of HF, respectively. (B) The calculated mass of PF_5_-Ubq is 9667.83 Da, which is same as the observed major peak. (C) The calculated mass of PF_5_-GB1 (after loss of the N-terminal methionine) is 8539.13 Da. The series peaks at – 20 Da and – 40 Da again likely correspond to the loss of one and two molecules of HF.

**Hydrolysis of PF_5_-Ubq to PO_3_-Ubq**

PF5-ubiquitin (100 µM, 95 µL) solution was adjusted to 5% perchloric acid (PCA) and allowed to react at 4 °C for 5 days. Afterwards the solution was adjusted to 5% trichloroacetic acid (TCA) and the ubiquitin allowed to precipitate at 4°C for 2 h. The ubiquitin was collected by centrifugation at 13,000 X g for 15 min at 4°C. The pellets were washed twice in acetone and dried under reduced pressure. The reaction product was analyzed via an ESI-Q-TOF iFunnel mass spectrometer. The cleavage of the His-Tag and one C-terminal serine residue were observed.

HRMS (ESI): [M^-^+H^+^] calculated for C_383_H_633_N_105_O_119_S_1_F_2_P: 8713.83 Da, found: 8714.54 Da (deconvoluted mass)


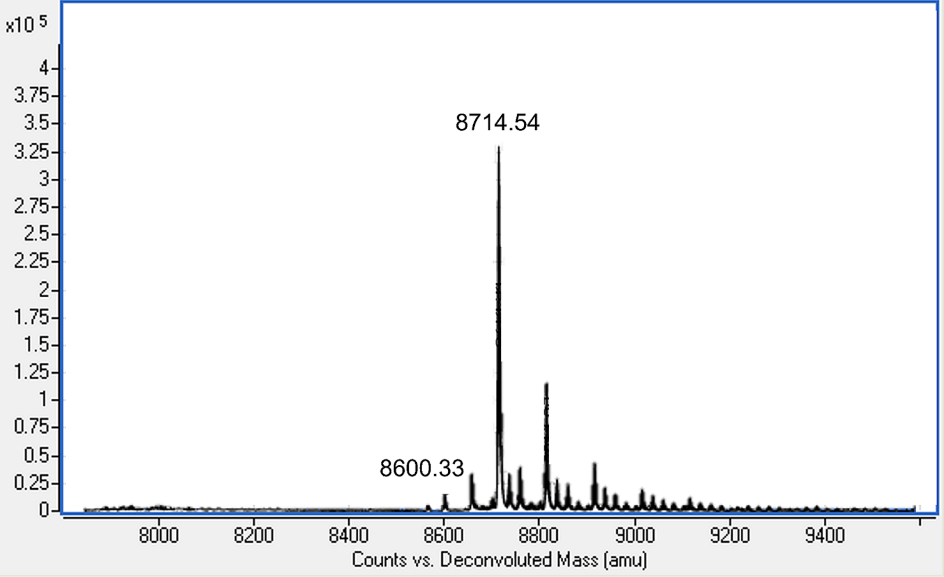


**Figure S13**. Protein mass spectrometric analysis of PO_3_-Ubq formed after treatment of PF_5_-Ubq with 5% PCA for 5 d at 4 °C. Cleavage of the C-terminal His-tag and one serine residue was observed with an expected mass of 8713.83 Da and found mass of 8714.54 Da. A small amount of further cleaved product (loss of 2 C-terminal glycine residues) was also found with an expected mass of 8598.79 Da and found mass of 8600.33 Da.

**Phosphatase activity assays using DiFMUP as a substrate**

Phosphatase activities were determined using a previously established method.^[22]^ Briefly, all assay components were diluted in the assay buffer, which contained 50 mM MOPSO (pH = 7.0), 200 mM NaCl, 0.03% Tween-20. Tris-(2-carboxyethyl)-phosphine (TCEP) was added freshly prior to each measurement to an end concentration of 50 μM. 6,8-Difluoro-4-methylumbelliferyl phosphate (DiFMUP) was dissolved in DMSO and stored in aliquots at a concentration of 20 mM at -20 °C and diluted with buffer to an end concentration of 67 μM. For determining phosphatase activity of PF5-protein samples (Method A), the proteins (20 or 50 μM) were incubated with DiFMUP (final concentration 67 μM) for 30 s, centrifuged and then measured at 37 °C for 10 min.

For testing the inhibition of protein tyrosine phosphatases (Method B), the catalytic domain of PTP1B was purchased from Abcam (ab51277) and used without further purification. Aliquots were stored at -80 °C. The catalytic domain of human SHP2 (amino acids 225–541) was expressed in *E. coli* and purified according to literature.^[41]^ Aliquots were stored at -80 °C.

PTP1B and SHP2 were incubated with probes of interest for 15 min, then DiFMUP (final concentration 67 μM) was added and incubated for another 30 s. After centrifugation, samples were measured at 37 °C for 10 min. Phosphatase activity of single aliquots of PTP1B and SHP2 was checked prior to each measurement (Method A). Suitable phosphatase activity was usually observed at a concentration of 2.5 nM.


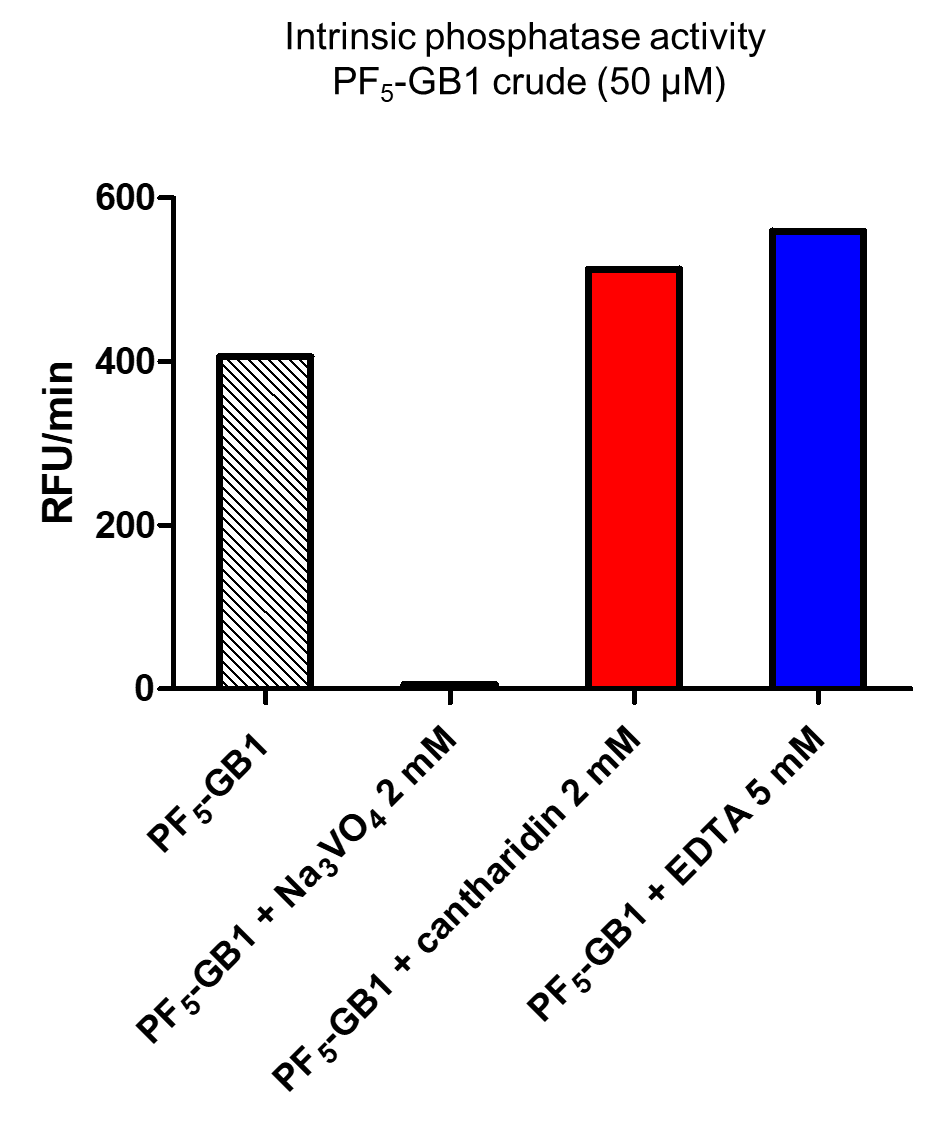


**Figure S14.** Intrinsic phosphatase activity of crude PF_5_-GB1 sample (Ni-NTA purified, 50 μM) measured in DiFMUP phosphatase assay (Method A) (RFU/min = 496.5) and after incubation with phosphatase inhibitors 2 mM Na_3_VO_4_ (RFU/min = 5.9), cantharidin (RFU/min = 513.2) and EDTA (RFU/min = 559.6) (single data points).

**SDS PAGE**

SDS-PAGE was performed to determine the purity of the expressed proteins. Samples were mixed with Laemmli sample loading buffer and heated to 95 °C for 5 min. Samples were then loaded into precast 14% Bis-Tris 1.0 mm minigels. Electrophoresis was performed at room temperature for approximately 90 min using a constant voltage (120 V) in a 192 mM glycine; 25 mM Tris; 0.1% SDS running buffer until the dye front reached the end of the 60 mm gel. Gels were washed three times in Millipore water for 5 min. SDS-PAGE gels were stained using RotiBlue (Roth# A152.1) over night. Gels were washed with Millipore water for 5 min, then destained. To make small protein amounts visible, silver staining was used. Briefly, gels were incubated with sodium thiosulfate pentahydrate (20 mg/100 mL) for 1 min, then washed three times with Millipore water. Following, silver nitrate solution (25 mL of 0.2 g/100 mL) and formaldehyde (19 μL of 37% aqueous solution) was added for 3 min. After another washing step with Millipore water, Na_2_CO_3_ solution (25 mL of 6 g/100 mL), formaldehyde (13 μL of 37% aqueous solution) and silver nitrate solution (3.3 mL of 0.2 g/100 mL) was added until sufficient staining was observed (usually 10 min). To stop the reaction, 10% acetic acid was added.

**Size exclusion chromatography**

The final purification of the PF_5_-Ubq and PF_5_-GB1 was performed on an ÄKTA pure^TM^ chromatography system using a Superdex^TM^ 75 Increase 10/300 GL column (product number 29148721 by Cytiva, bed dimensions 10 x 300 mm, particle size d_50V_ ~ 9 μM). 100 mM NH_4_HCO_3_ buffer was used as mobile phase with a flow rate of 0.4 mL/min. The proteins were eluted in 2.5 CV after injection (**Figure S15**). Peaks of interest were analyzed via SDS-PAGE. (**Figure S16**).

**
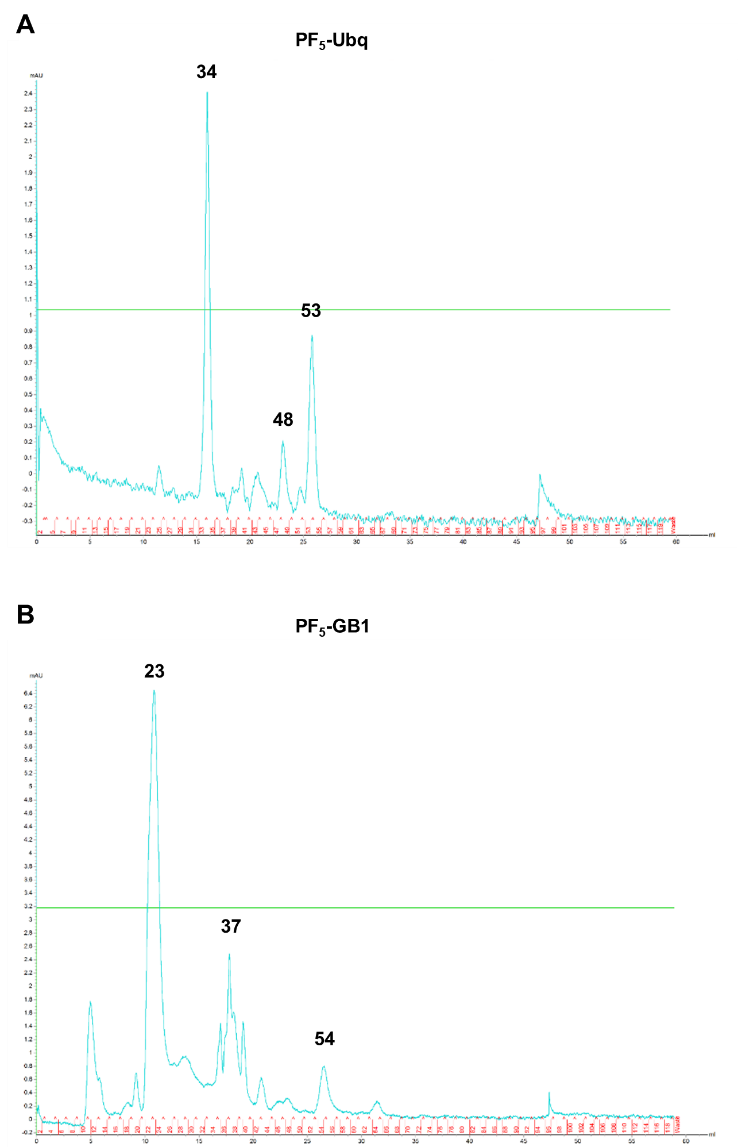
**

**Figure S15.** Purification of crude PF_5_-Ubq (A) and PF_5_-GB1 (B) using size-exclusion chromatography.

**
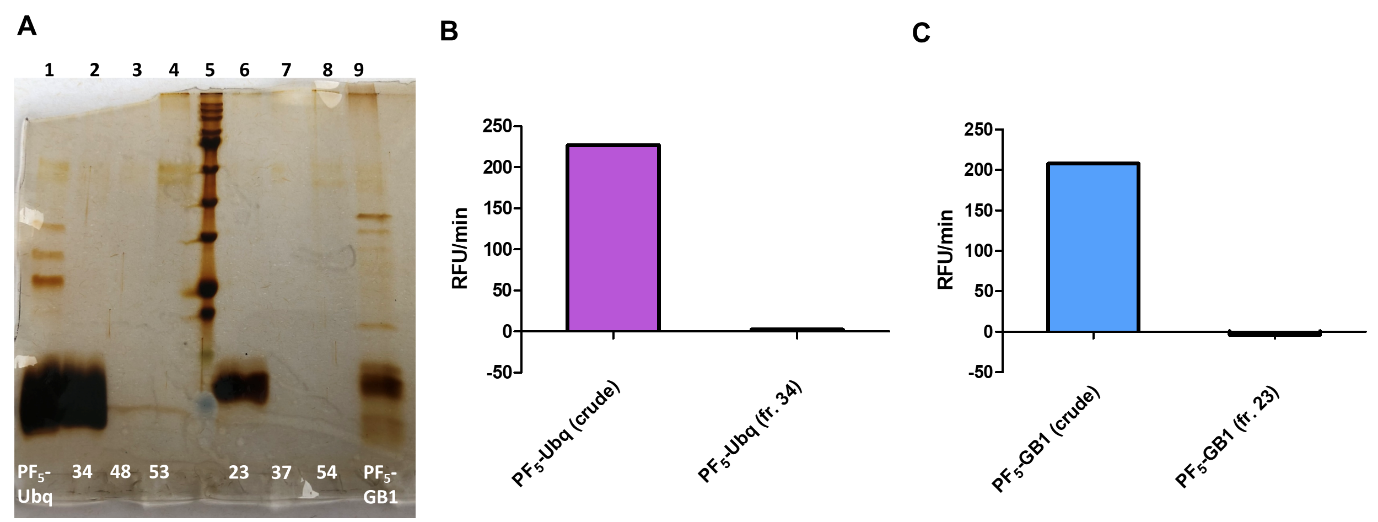
**

**Figure S16.** SDS-PAGE and intrinsic phosphatase activities of PF_5_-protein samples prior and after purification via SEC. (A) SDS-PAGE coloured with silver staining, Ni-NTA purified protein samples on lanes 1 (PF_5_-Ubq) and 9 (PF_5_-GB1), SEC-purified fractions with strongest UV absorption on lanes 2, 3 and 4 (PF_5_-Ubq) and lanes 6, 7 and 8 (PF_5_-GB1), protein marker on lane 5; (B) Intrinsic phosphatase activity of PF_5_-Ubq prior to SEC (crude) and after SEC (fr. 34) showing no residual phosphatase activity of the purified protein (DiFMUP phosphatase assay, 20 μM protein, method A, single data point); (C) Intrinsic phosphatase activity of PF_5_-GB1 prior to SEC (crude) and after SEC (fr. 23) showing no residual phosphatase activity of the purified protein (DiFMUP phosphatase assay, 20 μM protein, method A, single data point).

**Pull-down of interacting proteins from E. coli cell lysate and peptide analysis after tryptic digest**

Desalted PF_5_-Ubq, purified by immobilized metal ion affinity chromatography (IMAC) as described above, was loaded onto a 1 mL His GraviTrap column (Cytiva, USA). Pull-down assays were performed by flowing clarified 10 mL of *E. coli* (B95) cell lysate. After washing with three column volumes of buffer A, proteins were eluted with elution buffer (same as buffer A but with 500 mM imidazole) and loaded onto an SDS-PAGE. Gel slices were excised across two molecular weight regions: a band corresponding to Ubq (~8 kDa) to 30 kDa, and from 30 kDa to the top of the resolving gel. Gel pieces were processed for in-gel tryptic digestion following the protocol by Shevchenko et al.^[42]^ For a negative control of non-specifically binding (NSB) proteins, the same protocol was followed, without the loading of the PF_5_-Ubq onto the column. Great care was taken to ensure that identical volumes were used of the samples from PF_5_-Ubq samples and NSB protein samples.

Peptides were analyzed by data-dependent LC-MS/MS using a Thermo Orbitrap Fusion ETD mass spectrometer coupled to a Dionex UltiMate 3000 RSLC nano LC system via a Nanospray Flex nano-ESI ion source (Thermo Fisher Scientific). Separation was performed on an in-house packed C18 column (1.9 µm ReproSil-Pur 120 C18-AQ) using a 120 min gradient. MS1 scans were acquired in the Orbitrap at 120K resp;itopm (375-1500 m/z), followed by MS2 CID scans in the ion trap. Data were acquired in positive ion mode with a 2.3 kV spray voltage.

Raw files were converted to mzml using msConvert^[43]^ and analyzed using MaxQuant^[44]^, searching against the *Escherichia coli* (strain K12) UniProt reference proteome. Label-free quantitation (LFQ) was enabled. Oxidation (M) and N-terminal acetylation were set as variable modifications. A 1% false discovery rate was applied at peptide spectrum match and protein levels.

Identified peptides were organized into groups of their associated protein and analyzed in Andromeda^[45]^. The difference of the integrated intensities of the protein signals were then determined, producing a short list of 20 proteins that were enriched in the PF_5_CF_2_Phe pulldown sample, relative to the NSB sample where there was no corresponding protein in the NSB list, the intensity in the PF_5_CF_2_Phe list was used. The obtained list was filtered for proteins that have been annotated to be involved in phosphate-dependent pathways (**Table S3**). HisB was identified with an enrichment of 7.41 x 10^7^ for PF5-Ubq compared to NSB, supporting the hypothesis of potential binding of HisB to PF_5_-proteins.

**Table S6.** List of enriched phosphate-pathway related proteins from a pull down assay and subsequent LC-MS analysis. Out of these proteins, only HisB is known to cleave DiFMUP.^[27]^

| UniProt ID | Protein name | ΔIntensity | Unique peptides | Sequence coverage |
| --- | --- | --- | --- | --- |
| P0AG24 | Bifunctional (p)ppGpp synthase/hydrolase SpoT | 3.04E+08 | 6 | 0.15 |
| P06987 | Histidine biosynthesis bifunctional protein HisB | 7.41E+07 | 5 | 0.21 |
| P0A717 | Ribose-phosphate pyrophosphokinase | 1.11E+07 | 2 | 0.08 |
| P0A722 | Acyl-[acyl-carrier-protein]-UDP-N-acetylglucosamine O-acyltransferase | 1.09E+08 | 1 | 0.03 |
| P0A7B1 | Polyphosphate kinase | 1.13E+07 | 1 | 0.02 |

**Identification of proteins in crude protein samples by liquid chromatography-mass spectrometry (LC-MS)**

An in-solution digest of a phosphatase activity containing sample of PF_5_-GB1 was performed using the iST sample preparation kit from Preomics (product code P.O.00001). The peptide eluate was dried by vacuum centrifugation. For LC-MS analysis, the peptides were dissolved in 20 μl of 0.05% trifluoroacetic acid (TFA) with 5% acetonitrile, and 6 μl were analyzed by an Ultimate 3000 reverse-phase capillary nano liquid chromatography system connected to an Orbitrap Q Exactive HF mass spectrometer (Thermo Fisher Scientific) as described. Samples were injected and concentrated on a trap column (PepMap100 C18, 3 μm, 100 Å, 75 μM i.d. x 2 cm; Thermo Fisher Scientific) equilibrated with 0.05% TFA in water. After switching the trap column in-line, LC separations were performed on a reverse-phase column (Acclaim PepMap100 C18, 2 μm, 100 Å, 75 μm i.d. x 50 cm, Thermo Fisher Scientific) at an eluent flow rate of 300 nl/min. The mobile phase A contained 0.1% formic acid in water, and the mobile phase B contained 0.1% formic acid in 80% acetonitrile/20% water. The column was pre-equilibrated with 5% mobile phase B, followed by an increase of 5–44% mobile phase B in 70 min. The mass spectra were acquired in a data-dependent mode utilizing a single MS survey scan (*m*/*z* 300 - 1,650) with a resolution of 60,000, and MS/MS scans of the 15 most intense precursor ions with a resolution of 15,000 and a normalized collision energy of 27. The isolation window of the quadrupole was set to 1.4 *m*/*z*. The dynamic exclusion time was set to 20 s and automatic gain control was set to 3x10^6^ and 1x10^5^ for MS and MS/MS scans, respectively.

Data processing and identification of proteins was performed using the Mascot software package (Mascot Server version 2.7, Mascot Daemon version 3.0, Matrix Science). Processed spectra were searched against the SwissProt database using the taxonomy *E. coli* (23,266 sequences, June 2024). A maximum of two missed cleavages was allowed and the mass tolerance of precursor and sequence ions was set to 10 ppm and 0.02 Da, respectively. Methionine oxidation and acetylation no protein N-terminus were set as variable modifications. Cysteine carbamidomethylation was set as fixed modification. A significance threshold of 0.05 was used as a cut-off.

Table S4 shows the TOP20 most abundant proteins identified by LC-MS. Beside common impurities of IMAC purification (e.g. SlyD) the histidine biosynthesis bifunctional protein (HisB) was identified with 17 significant peptides and a high sequence coverage of 54 % (**Table S7**). HisB natively cleaves the phosphate group of L-histidinol phosphate^[26]^, it might be responsible for the intrinsic phosphatase activity of the expressed proteins.

**Table S7.** Results from LC-MS analysis of trypsinized sample of phosphatase activity containing PF_5_-GB1 with UniProt accession code. Score and exponentially modified protein abundance index (emPAI) are used as estimate for abundance. HisB is highlighted in yellow, which is the only found protein with reported phosphatase activity.

| Accession | Score | Mass | Num. of significant matches | Sequence coverage | emPAI |
| --- | --- | --- | --- | --- | --- |
| SLYD_ECOLI | 23548 | 21182 | 706 | 0.70 | 13.92 |
| GLMS_ECOLI | 22599 | 67081 | 477 | 0.78 | 15.08 |
| FUR_ECOLI | 10381 | 17012 | 248 | 0.67 | 13.02 |
| KPYK2_ECOLI | 8643 | 51553 | 179 | 0.71 | 23.76 |
| CRP_ECOLI | 7724 | 23796 | 197 | 0.84 | 124.62 |
| RS15_ECO24 | 7686 | 10263 | 221 | 0.70 | 50.45 |
| PURU_ECOL6 | 6716 | 32072 | 178 | 0.65 | 9.07 |
| SDHA_ECOLI | 5417 | 65008 | 144 | 0.57 | 5.74 |
| FADA_ECOK1 | 4757 | 41196 | 129 | 0.70 | 23.67 |
| TRMB_ECOHS | 4239 | 27404 | 132 | 0.79 | 16.24 |
| RAPZ_ECOBW | 4190 | 32586 | 116 | 0.82 | 55.62 |
| YEIE_ECOLI | 3779 | 32874 | 68 | 0.80 | 9.81 |
| EFTU1_ECO24 | 3491 | 43427 | 85 | 0.63 | 9.78 |
| HFQ_ECOBW | 3149 | 11160 | 70 | 0.51 | 16.81 |
| MTNN_ECOBW | 3126 | 24624 | 68 | 0.90 | 7.71 |
| ARGE_ECOBW | 3111 | 42777 | 79 | 0.62 | 7.37 |
| AHPF_ECOLI | 2982 | 56484 | 79 | 0.66 | 7.35 |
| HIS7_ECOL6 | 2826 | 40619 | 86 | 0.54 | 13.01 |
| RSD_ECOBW | 2807 | 18288 | 76 | 0.40 | 4.95 |
| RS2_ECO27 | 2567 | 26784 | 79 | 0.71 | 9.00 |

**Molecular modeling**

**Generation of PF_5_-GB1, PF_5_-Ubq and PO_3_-Ubq**

PDB structures 1UBQ (Ubiquitin) and 2J52 (GB1) were downloaded from www.rcsb.org. The structures were manually prepared using the structure preparation tool in MOE^[46]^ and protonation was done using Protonate3D.^[47]^ The individual variants of GB1 and Ubq (Figure 3) were manually constructed in MOE by exchanging Glu18 or Thr17 with the PF_5_CF_2_Phe and PO_3_CF_2_Phe moieties. The Amber14 force field^[48]^ was used for energy minimization of the PF_5_ variants and MMFF95 force field^[49]^ for PO_3_ minimization.

**
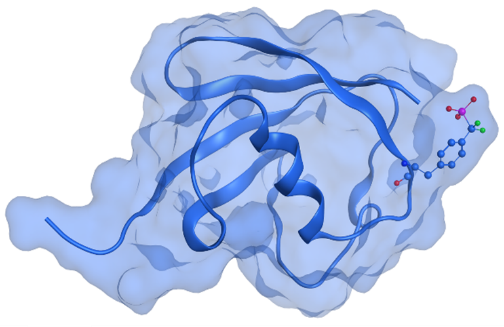
**

**Figure S17.** Protein model of PO_3_-Ubq built from the PDB structure 1UBQ in MOE.

**Protein-protein docking and modeling of PF_5_- and PO_3_-proteins**

PDB structures 1Q1M (PTP1B) and 2SHP (SHP2) were downloaded from www.rcsb.org. The structures were manually prepared using the structure preparation tool in MOE^[46]^ and protonation was done using Protonate3D.^[47]^ 100 docking poses were created using the protein-protein docking tool with standard settings. PTP1B and SHP2 were defined as receptor atoms and GB1 and Ubq as ligand atoms. The specific receptor site in PTP1B was defined as all residues which are in 4.5 Å proximity to the co-crystallized ligand.^[50]^ For SHP2, only the PTP domain was used for docking and the active site was defined according to described key PTP active site residues, Asn58, Arg278, Tyr279, Asp425, His426 and Arg465 respectively.^[51]^

Docking poses were selected via visual inspection since we first wanted to understand how Ubq and GB1 can in theory bind to the two phosphatases and why the enzyme activities was differently influenced by GB1 and Ubq variants. In addition, the protein-protein docking algorithm was found to be parametrized for native proteins only and not for PF_5_ or PO_3_ variants. Therefore, we manually changed Thr17 in GB1 and Glu18 in Ubq to the PF_5_CF_2_Phe or PO_3_CF_2_Phe moieties and checked if the variant residues would fit sterically into the binding site and could interact with the active site residues of PTP1B or SHP2.


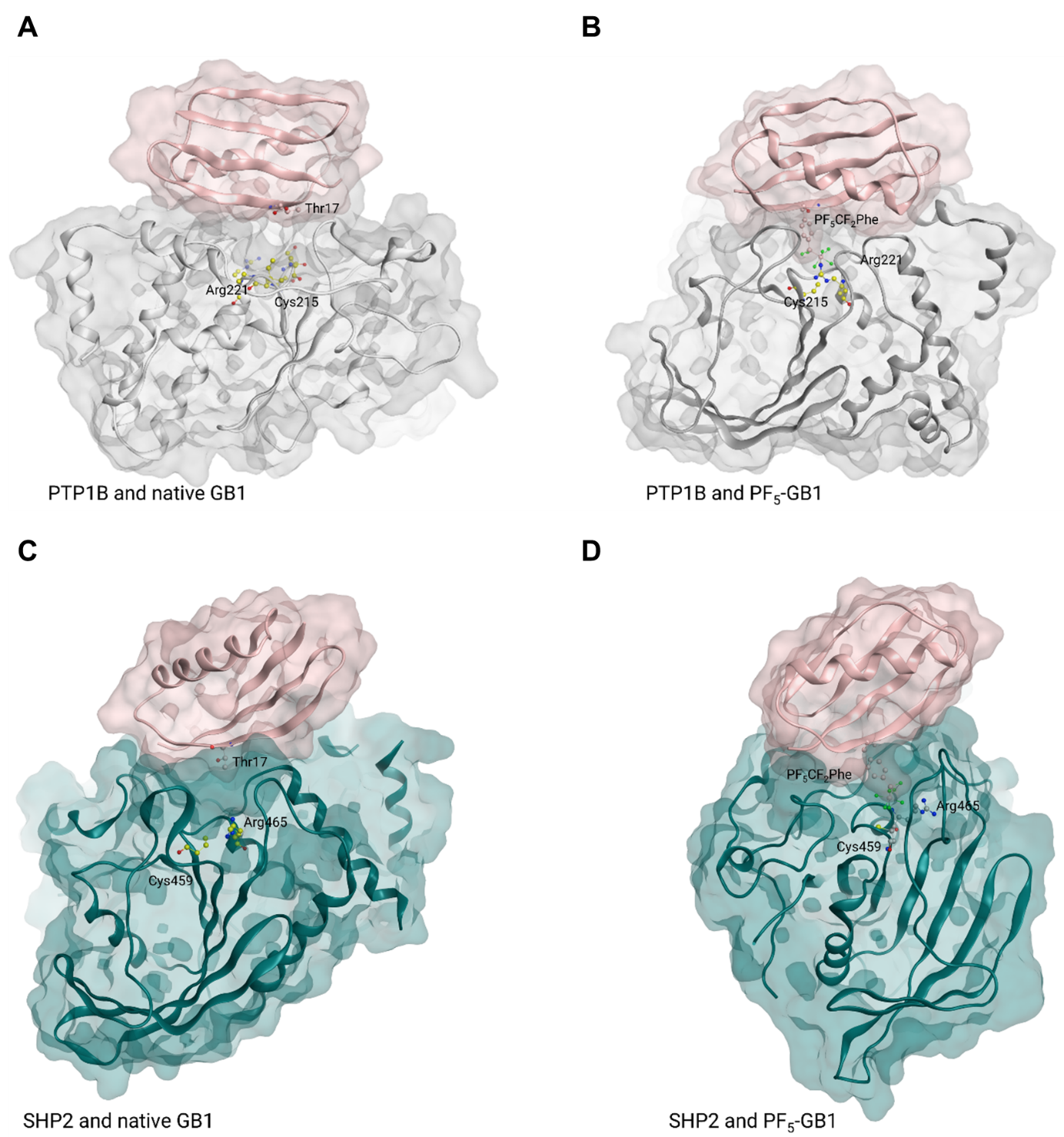


**Figure S18.** Protein-protein docking of GB1 variants with PTP1B and SHP2. The biological data indicated that PF_5_-GB1 binds to PTP1B and SHP2 in a way which blocks the active site, in contrast to native GB1. Therefore, we selected docking poses in which Thr17 was found in close proximity to the active site (**Figure S18A** for PTP1B, **Figure S18C** for SHP2). To investigate potential interactions of the pentafluorinated variants, Thr17 was manually changed to a PF_5_CF_2_Phe in MOE and the complex was then minimized using the standard Amber14 force field.^[46]^ We found that the PF_5_CF_2_Phe residue fits well into the active sites of PTP1B and SHP2. The PF_5_CF_2_Phe moiety was found to be near the positively charged Arg221 (**Figure S18B** for PTP1B) and Arg465 (**Figure S18D** for SHP2), indicating a salt bridge at that position. No atom clashes were detected after the minimization step.


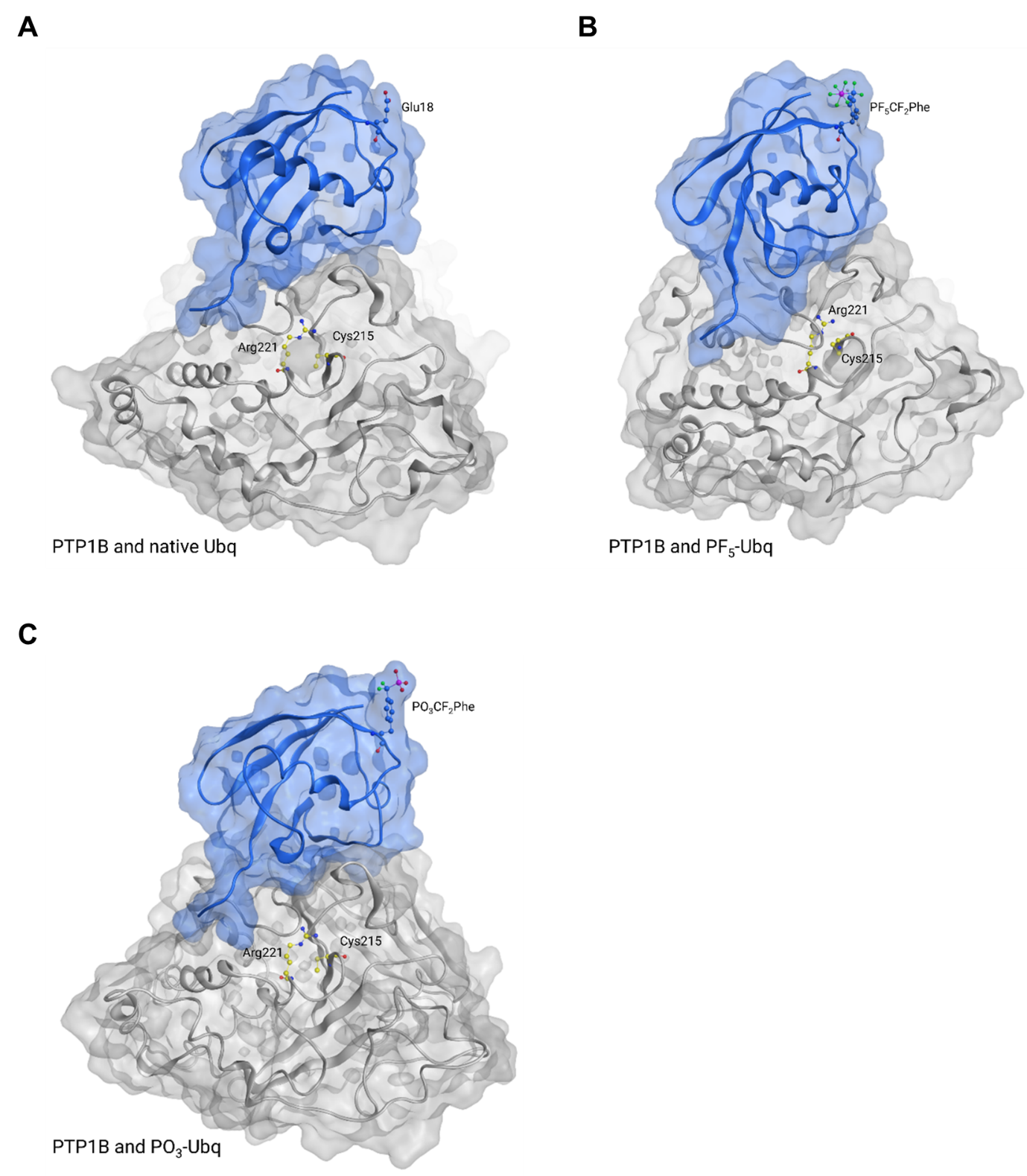


**Figure S19.** Protein-protein docking of Ubq variants with PTP1B. For PTP1B native Ubq, PF_5_-Ubq and PO_3_-Ubq did not lead to enzyme inhibition, which would indicate that none of the three proteins is able to bind to the active site of the phosphatase. Indeed, none of the 100 docked poses showed binding of Ubq in proximity to the catalytic center. The reason for this is likely the bulky shape of Ubq which hinders the approach to the active site. To visualize the protein-protein interaction, we used the docking pose with the highest score (**Figure S19A**) and manually changed Glu18 into a PF_5_CF_2_Phe (**Figure S19B**) or PO_3_CF_2_Phe residue (**Figure S19C**) before we minimized the complexes, using the Amber14 force field for the PF_5_-variant and the MMFF94 force field for the PO_3_-variant.

**
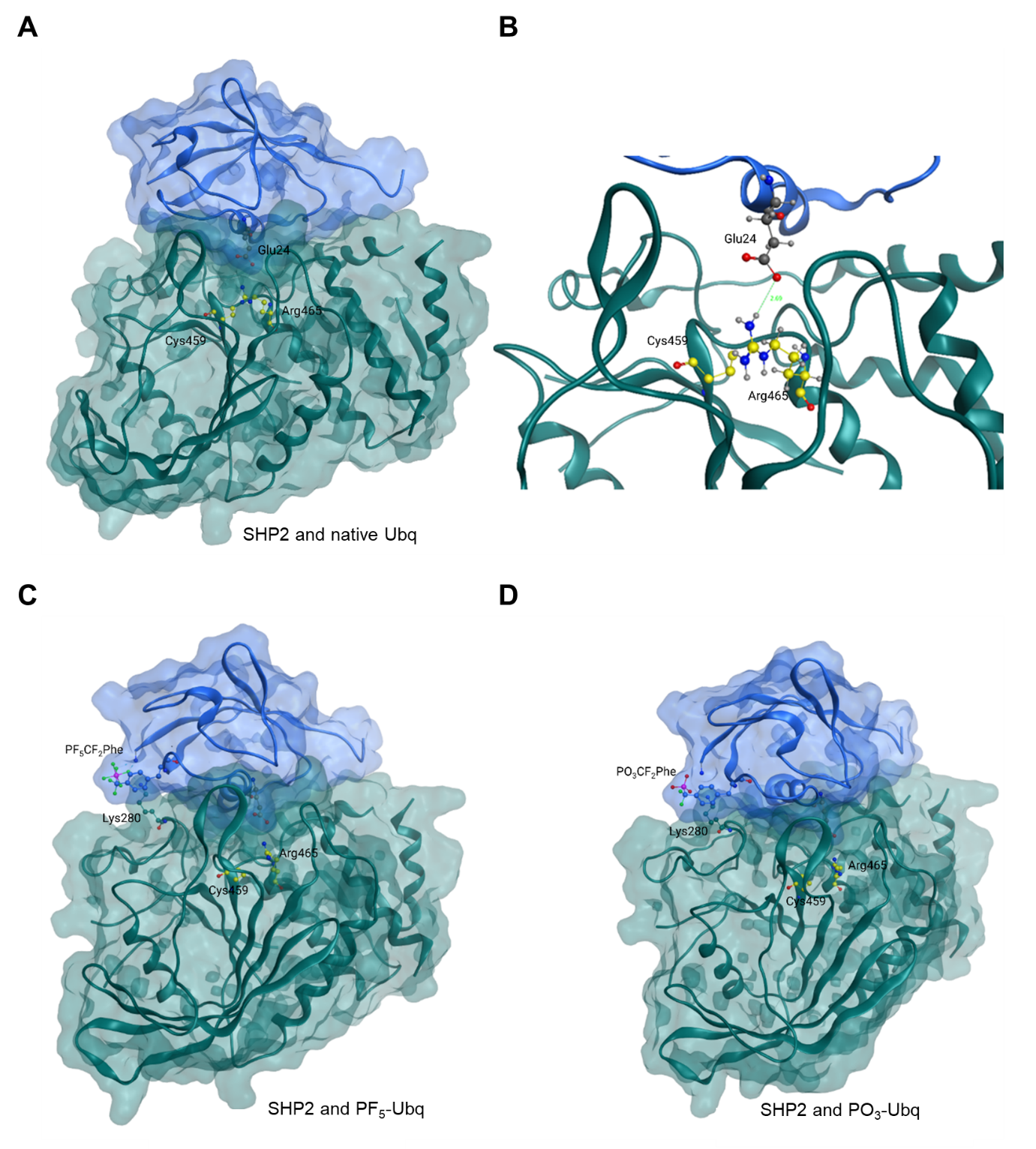
Figure S20.** The biological activities of the different Ubq variants binding to SHP2 are similar with a remaining enzyme activity of approximately 50%. Therefore, it seems likely that an invariant glutamic or aspartic acid binds to the active site which results in the observed decreased enzyme activity. The visual pose inspection of all docked poses indicated that Glu24 could potentially interact with the active site (**Figure S20A**). After energy minimization of the protein-protein complex, Glu24 is 2.69 Å from Arg465 of the active site of the enzyme (**Figure S20B**). This distance indicates a close-range salt-bridge and a hydrogen bond between the two residues. In this position, PF_5_ (**Figure S20C**) and PO_3_ (**Figure S20D**) would reach into the solvent, showing no interaction with the active site of the protein, however, their negative charge could be stabilized by the positively charged Lys280 of PTP1B.

**NMR spectra**

**
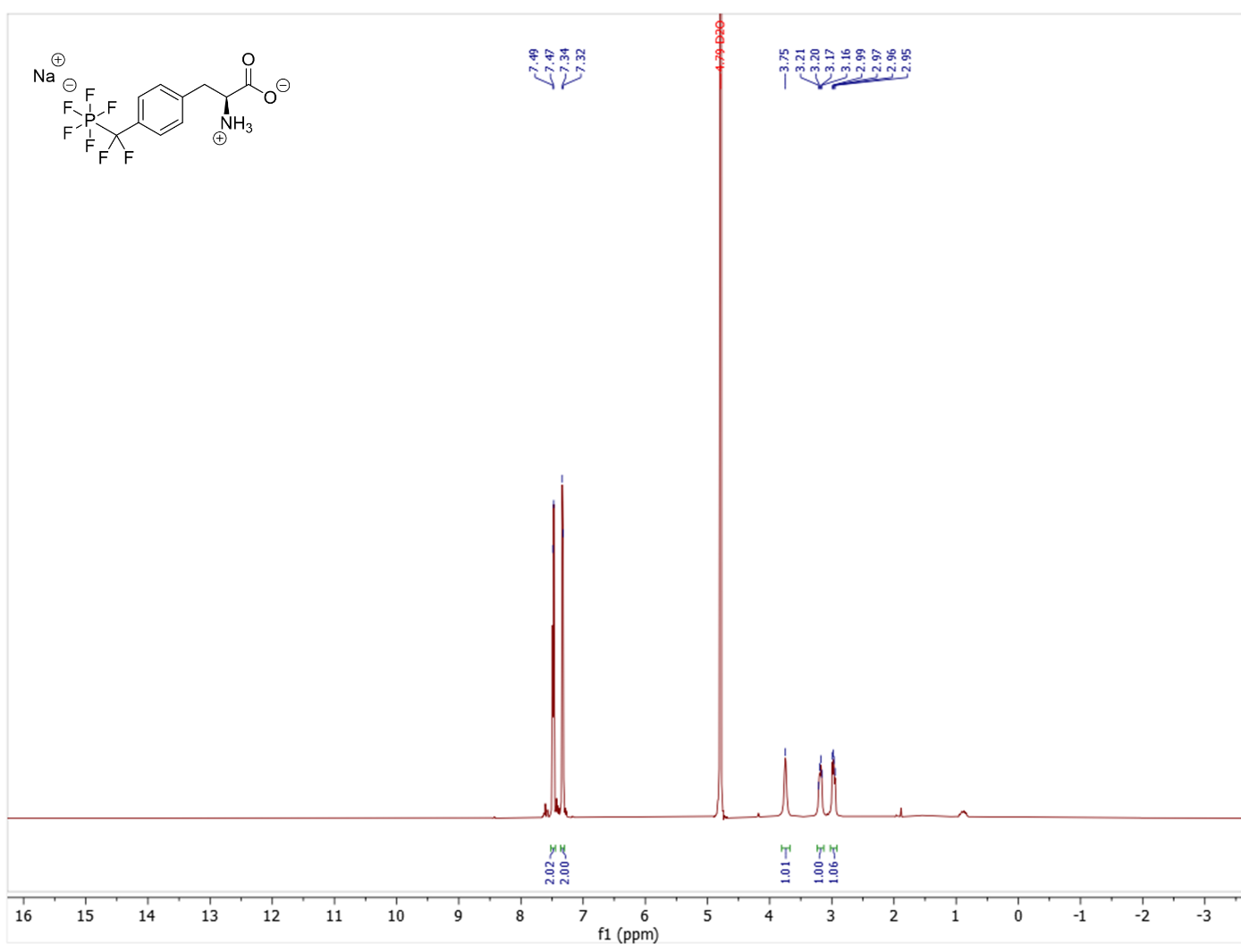
**

**Figure S21.** ^1^H NMR (500 MHz, D_2_O) of **3**


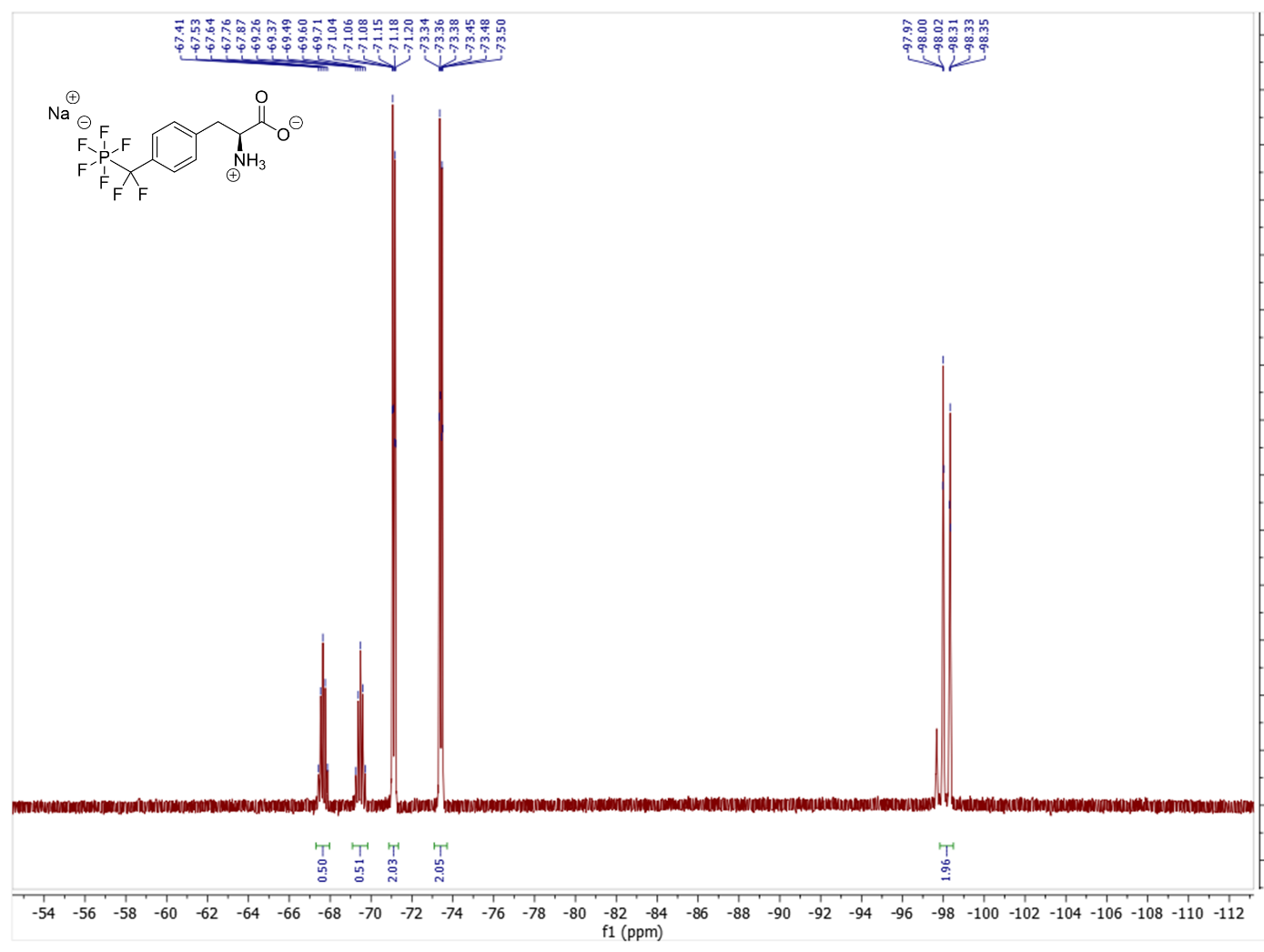


**Figure S22.** ^19^F NMR (376 MHz, D_2_O) of **3**


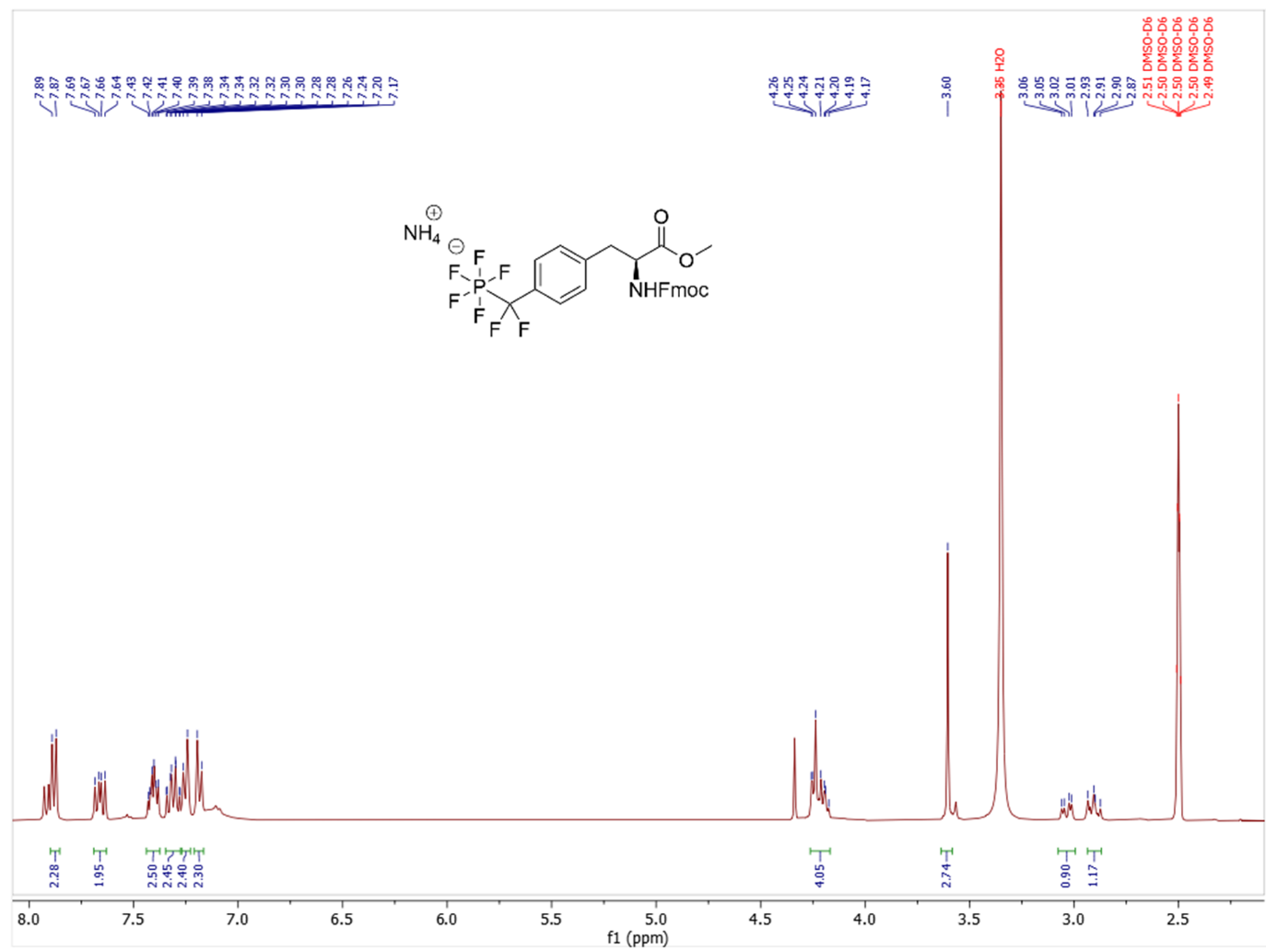


**Figure S23.** ^1^H NMR (400 MHz, DMSO-D6) of **6**


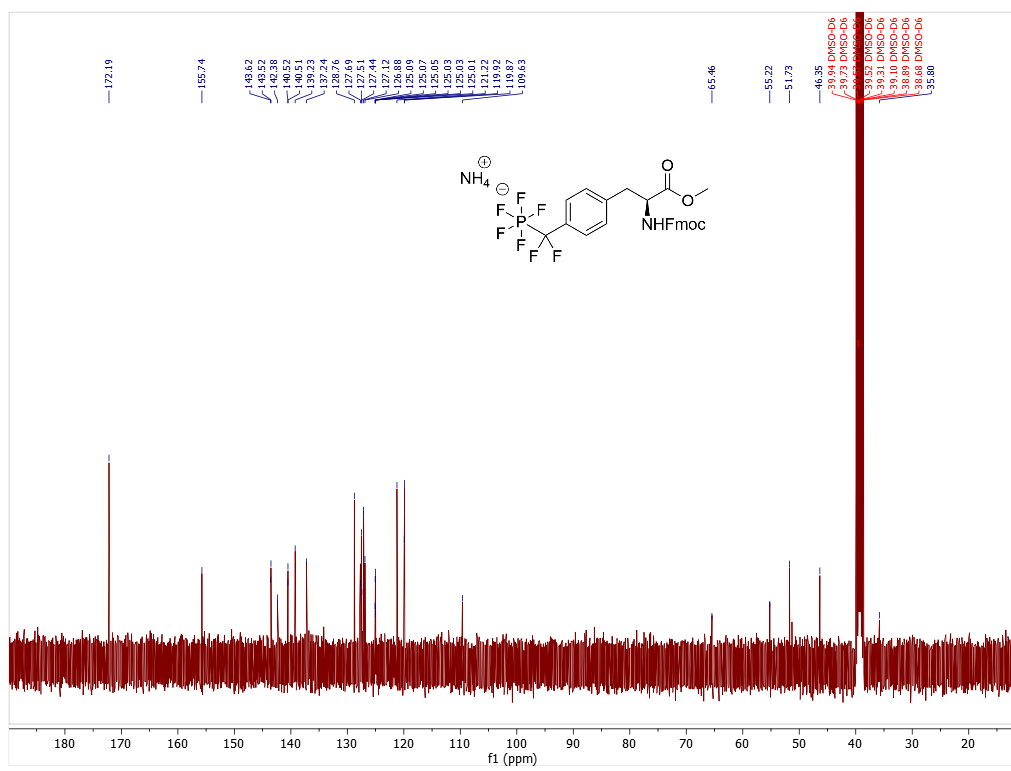


**Figure S24.** ^13^C NMR (100 MHz, DMSO-D6) of **6**


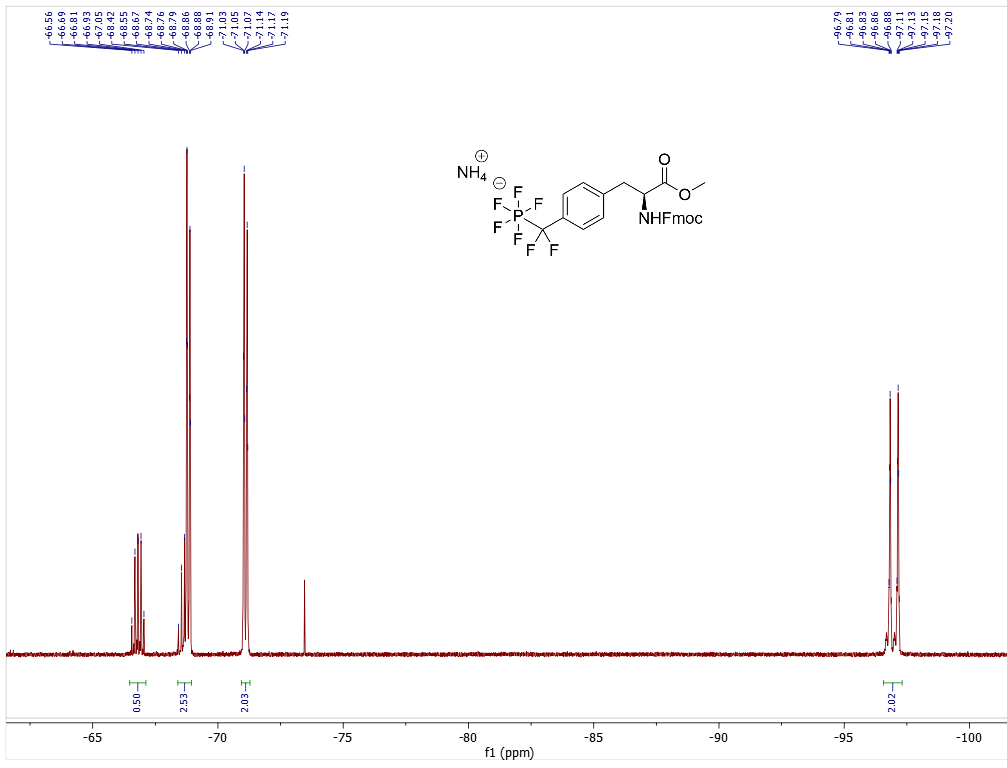


**Figure S25.** ^19^F NMR (376 MHz, DMSO-D6) of **6**


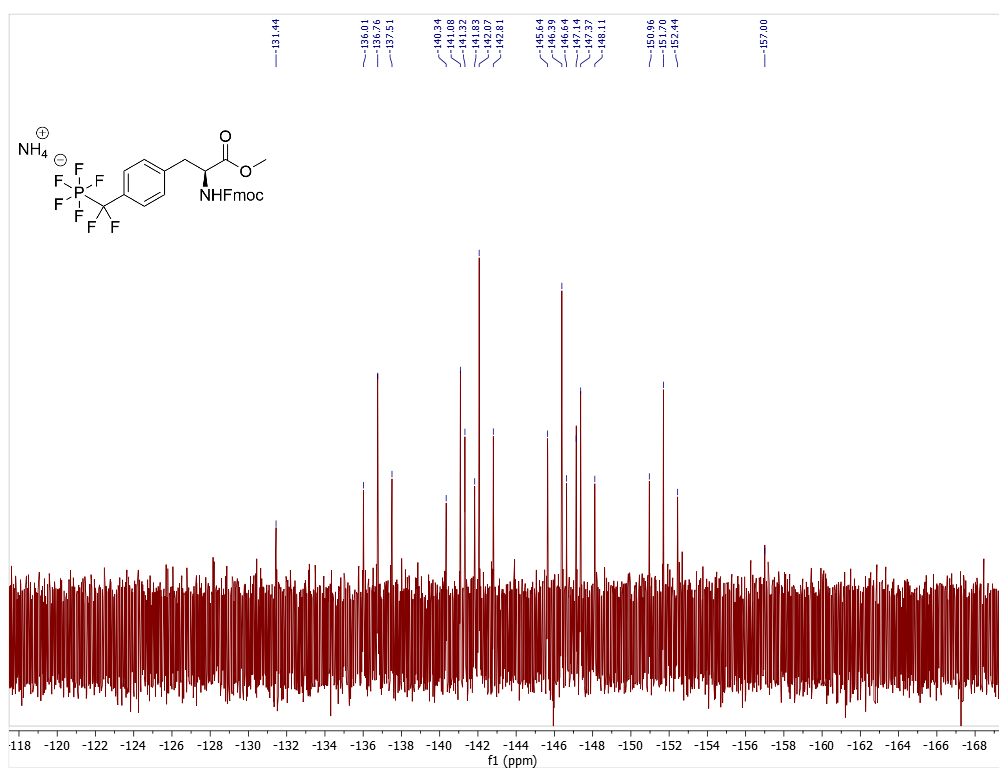


**Figure S26.** ^31^P NMR (162 MHz, DMSO-D6) of **6**


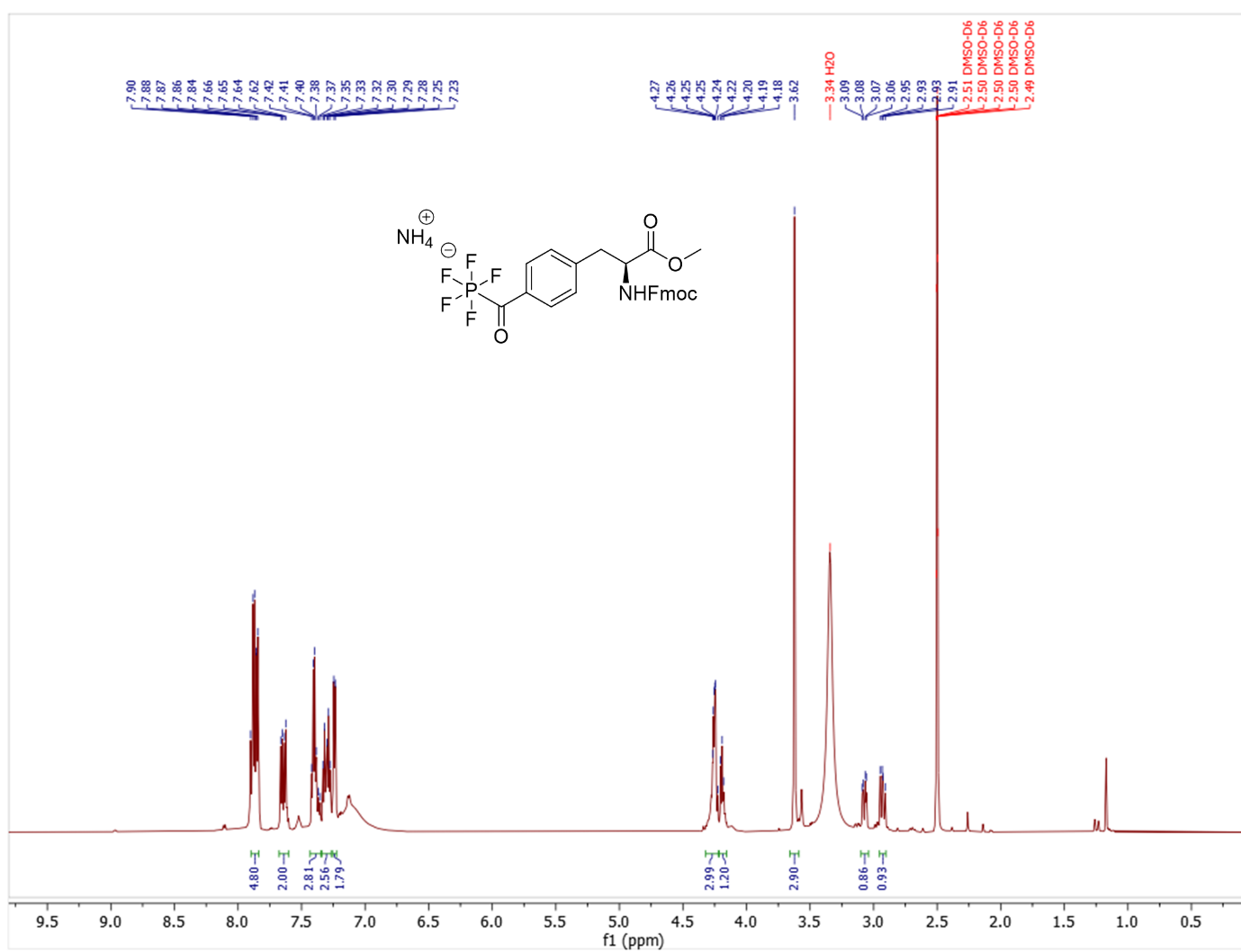


**Figure S27.** ^1^H NMR (600 MHz, DMSO-D6) of **7**

**
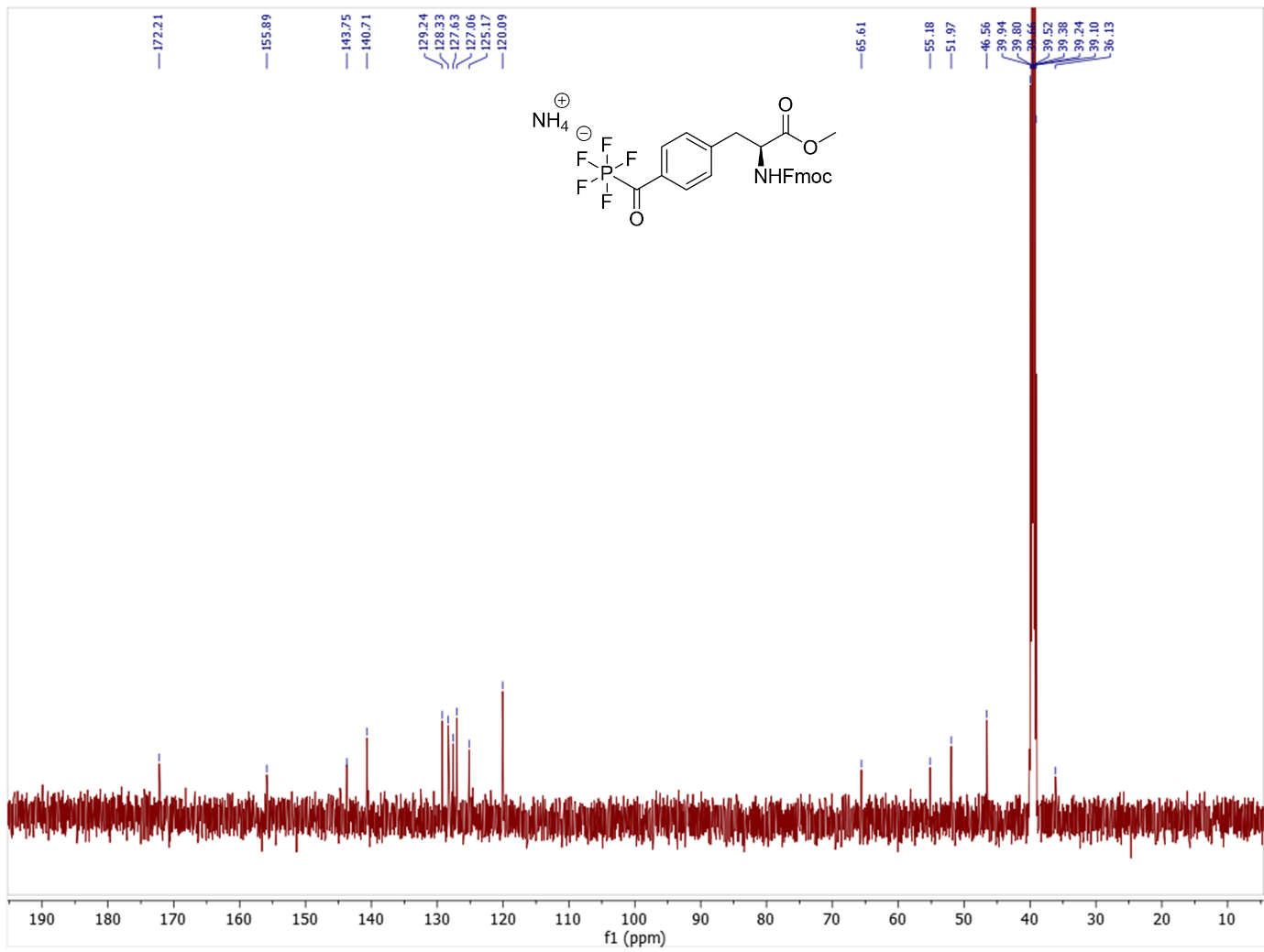
**

**Figure S28.** ^13^C NMR (151 MHz, DMSO-D6) of **7**


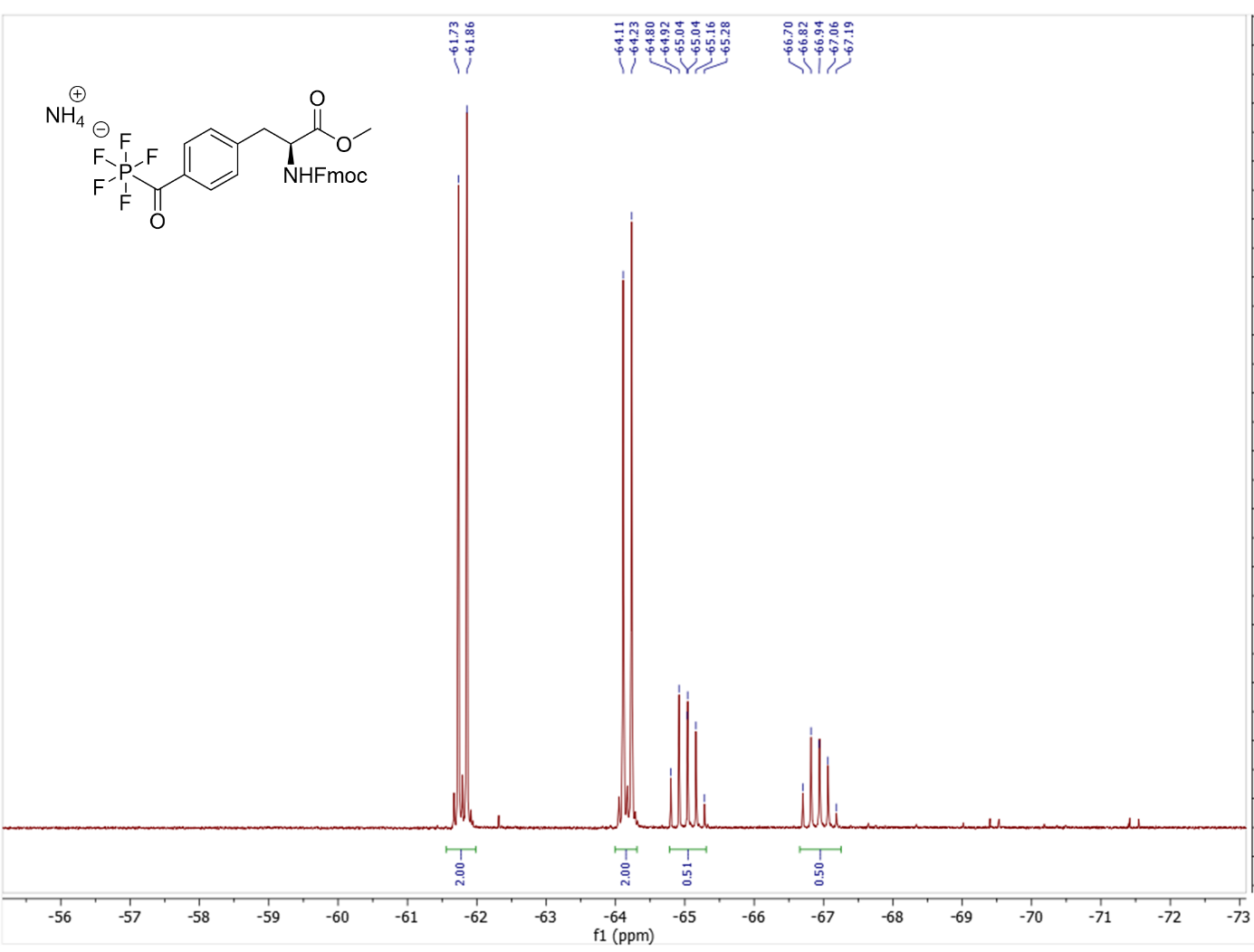


**Figure S29.** ^19^F NMR (376 MHz, DMSO-D6) of **7**


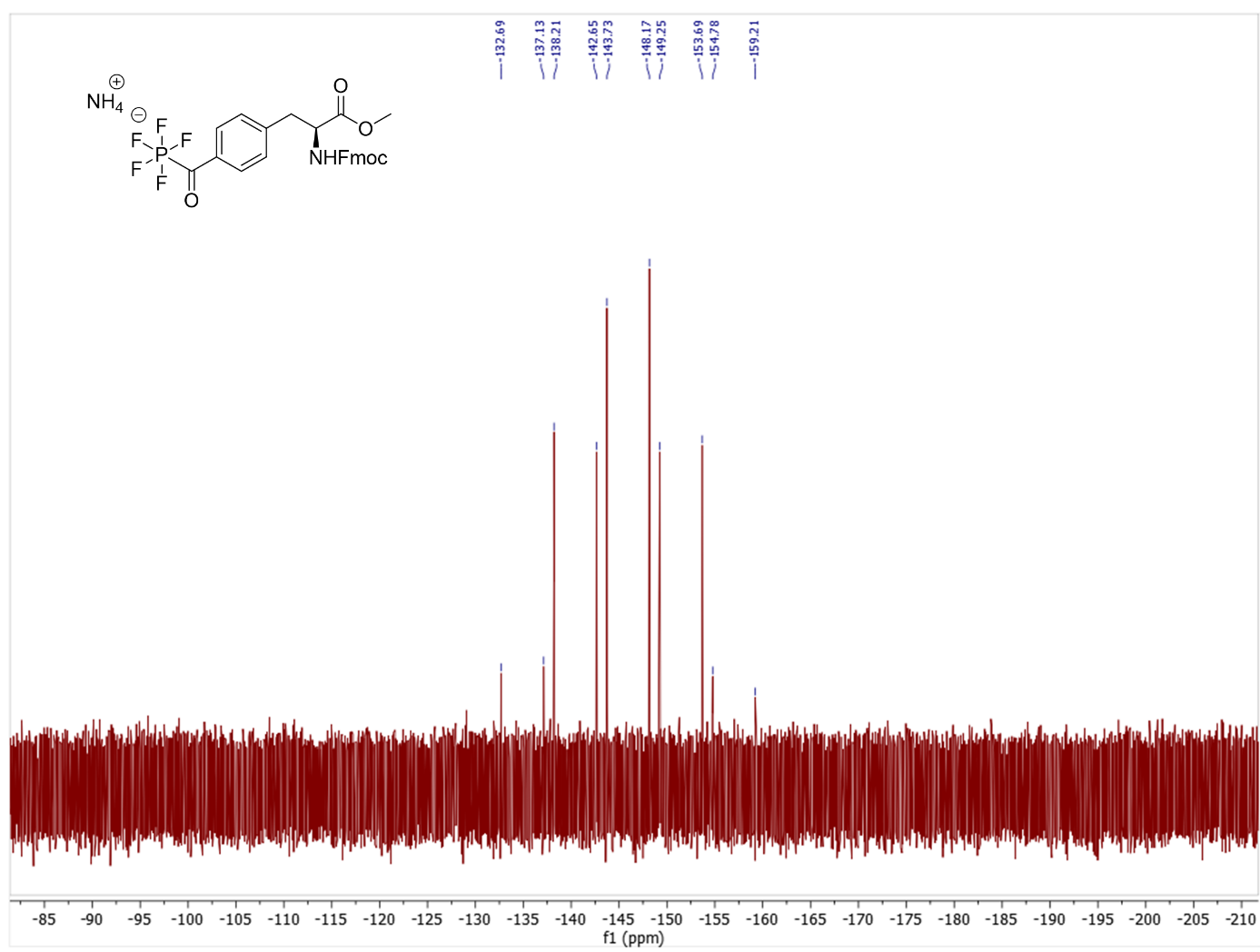


**Figure S30.** ^31^P NMR (162 MHz, DMSO-D6) of **7**

**Supplementary References**

[30] Schrödinger, LLC. “The PyMOL Molecular Graphics System, Version 1.8”. **2015**.

[31] B. de Souza, *Angew. Chem. Int. Ed.* **2025**, *64*, e202500393.

[32] S. Ehlert, M. Stahn, S. Spicher, S. Grimme, *J. Chem. Theory Comput.* **2021**, *17*, 4250-4261.

[33] A. V. Marenich, C. J. Cramer, D. G. Truhlar, *J. Phys, Chem. B.* **2009**, *113*, 6378-6396.

[34] F. Neese, *Interdiscip. Rev.: Comput. Mol. Sci.* **2012**, *2*, 73-78.

[35] C. Bannwarth, E. Caldeweyher, S. Ehlert, A. Hansen, P. Pracht, J. Seibert, S. Spicher, S. Grimme, *WIREs Comput. Mol. Sci.* **2020**, *11*, e1493.

[36] a) G. Schaftenaar, J. H. Noordik, *J. Comput. Aided Mol. Des.* **2000**, *14*, 123-34. b) G. Schaftenaar, E. Vlieg, G. Vriend, *J. Comput. Aided Mol. Des.* **2017**, *31*, 789-800.

[37] H. Qianzhu, E. H. Abdelkader, I. D. Herath, G. Otting, T. Huber, *ACS Sens.* **2022**, *7*, 44–49.

[38] D. D. Young, T. S. Young, M. Jahnz, I. Ahmad, G. Spraggon, P. G. Schultz, *Biochemistry* **2011**, *50*, 1894–1900.

[39] T. S. Young, I. Ahmad, J. A. Yin, P. G. Schultz, *J. Mol. Biol.* **2010**, *395*, 361–374.

[40] J. Abramson et al. *Nature* **2024**, *630*, 493-500.

[41] M. Tiemann, E. Nawrotzky, P. Schmieder, L. Wehrhan, S. Bergemann, V. Martos, W. Song, C. Arkona, B. G. Keller, J. Rademann, *Chem. Eur. J.* **2022**, *28*, e202201282.

[42] A. Shevchenko, H. Tomas, J. Havli, J. V. Olsen, M. Mann, *Nat. Protoc.* **2006**, *1*, 2856-2860.

[43] R. Adusumilli, P. Mallick, *Methods Mol Biol.* **2017**, *1550*, 339-368.

[44] S. Tyanova, T. Temu, J. Cox, *Nat. Protoc.* **2016**, *11*, 2301-2319.

[45] J. Cox, N. Neuhauser, A. Michalski, R. A. Scheltema, J. V. Olsen, M. Mann, *J. Proteome Res.* **2011**, *10*, 1794-1805.

[46] Molecular Operating Environment, Version 2022.02, Chemical Computing Group ULC, 910-1010 Sherbrooke St. W., Montreal, QC H3A 2R7, **2025**.

[47] P. Labute, *Proteins: Struct. Funct. Bioinf.* **2008**, *75*, 1-272.

[48] D. A. Case, V. Babin, J. Berryman et al., *University of California, San Francisco,* **2014**.

[49] T. A. Halgren, *J. Comput. Chem.* **1996**, *17*, 490-519.

[50] G. Liu, Z. Xin, Z. Pei, P. J. Hajduk, C. Abad-Zapatero, C. W. Hutchins, H. Zhao, T. H. Lubben, S. J. Ballaron, D. L. Haasch, W. Kaszubska, C. M. Rondinone, J. M. Trevillyan, M. R. Jirousek, *J. Med. Chem.* **2003**, *46*, 4232-4235.

[51] P. Hof, S. Pluskey, S. Dhe-Paganon, M. J. Eck, S. E. Shoelson, *Cell* **1998**, *92*, 441-450.
